# “Dual sensitive supramolecular curcumin nanoparticles” in “advanced yeast particles” mediate macrophage reprogramming, ROS scavenging and inflammation resolution for ulcerative colitis treatment

**DOI:** 10.1186/s12951-023-01976-2

**Published:** 2023-09-07

**Authors:** Xiaoqin Han, Ruifeng Luo, Shanshan Qi, Yanli Wang, Linxin Dai, Wenbiao Nie, Meisi Lin, Haoqi He, Naijing Ye, Chaomei Fu, Yu You, Shu Fu, Fei Gao

**Affiliations:** 1 State Key Laboratory of Southwestern Chinese Medicine Resources, Pharmacy School, Chengdu University of Traditional Chinese Medicine, 611130 Chengdu, China; 2 TCM Regulating Metabolic Diseases Key Laboratory of Sichuan Province, Hospital of Chengdu University of Traditional Chinese Medicine, 610072 Chengdu, China; 3 State Key Laboratory of Quality Research in Chinese Medicine, Institute of Chinese Medical Sciences, University of Macau, 999078 Taipa, Macau China

**Keywords:** Yeast cell wall microparticles, Macrophage targeting, Curcumin, pH/ROS dual-responsive, Ulcerative colitis, Supramolecular nanoparticles

## Abstract

Ulcerative colitis (UC) faces some barriers in oral therapy, such as how to safely deliver drugs to the colon and accumulate in the colon lesions. Hence, we report an advanced yeast particles system loaded with supramolecular nanoparticles with ROS scavenger (curcumin) to treat UC by reducing oxidative stress state and inflammatory response and accelerating the reprogramming of macrophages. In this study, the dual-sensitive materials are bonded on β-cyclodextrin (β-CD), the D-mannose (Man) is modified to adamantane (ADA), and then loaded with curcumin (CUR), to form a functional supramolecular nano-delivery system (Man-CUR NPs) through the host-guest interaction. To improve gastrointestinal stability and colonic accumulation of Man-CUR NPs, yeast cell wall microparticles (YPs) encapsulated Man-CUR NPs to form Man-CUR NYPs via electrostatic adsorption and vacuum extrusion technologies. As expected, the YPs showed the strong stability in complex gastrointestinal environment. In addition, the Man modified supramolecular nanoparticles demonstrated excellent targeting ability to macrophages in the *in vitro* cellular uptake study and the pH/ROS sensitive effect of Man-CUR NPs was confirmed by the pH/ROS-dual stimulation evaluation. They also enhanced lipopolysaccharide (LPS)-induced inflammatory model in macrophages through downregulation of pro-inflammatory factors, upregulation of anti-inflammatory factors, M2 macrophage polarization, and scavenging the excess ROS. Notably, in DSS-induced mice colitis model, Man-CUR NYPs can reduce the inflammatory responses by modulating TLR4/NF-κB signaling pathways, alleviate oxidative stress by Nrf2/HO-1 signaling pathway, promote macrophages reprogramming and improve the favorable recovery of the damaged colonic tissue. Taken together, this study not only provides strategy for “supramolecular curcumin nanoparticles with pH/ROS sensitive and multistage therapeutic effects” in “advanced yeast particles”, but also provided strong theoretical support multi-effect therapy for UC.

**Supplementary Information:**

The online version contains supplementary material available at 10.1186/s12951-023-01976-2.

## Introduction

As a chronic and recurrent disease of colitis, ulcerative colitis (UC) has developed into one of the most common intestinal diseases worldwide [1]. It is characterized by the ulceration of superficial mucosa in the rectum and colon, which is accompanied by rectal bleeding, diarrhea and weight loss, posing severe threat to human health and increasing the risk of cancer [2]. At present, its incidence is relatively high in North America and Europe, showing a clear upward trend in some Asian countries [3]. The mainstream therapeutic drugs used for UC treatment include aminosalicylic acid, steroids and immunosuppressants. However, their therapeutic activity is relatively week there is potential risk of toxicity [4]. In recent years, natural active ingredients have attracted widespread attention due to their multi-target and multi-channel therapeutic effects. It has been increasingly report showed that they are promising in the terms of UC treatment [5, 6]. However, there are two major challenges in the treatment of UC. One is to improve the bioavailability of active ingredients for the target cell. The other is to deliver active ingredients to the colon inflammatory site efficiently given that colon lesion is located at the distal of the gastrointestinal tract.

In recent years, the yeast cell wall microparticles (YPs) extracted from baker’s yeast *Saccharomyces cerevisiae* have been used for oral delivery of drugs or probiotics [7, 8]. Firstly, the sufficient rigidity of its peptidoglycan layer can protect the drug from passing through the gastric environment [9]. Secondly, it can be digested with the assistance of abundant intestinal microbiota around the colon lesion, which allows the release loaded drugs or NPs [10]. Thirdly, it features a hollow porous microsphere structure, which is conductive to drug loading [11]. In our previous studies, protein-loaded NPs were successfully encapsulated with YPs [12, 13]. However, there is still no report on the UC treatment carried out by using YPs to encapsulate functional supramolecular NPs (supra-NPs).

The oral micro/nano-drug delivery system shows a great potential for use in the treatment of UC [14]. In this respect, some nano delivery systems with active and passive targeting properties have been developed to improve the concentration of drugs in inflammatory target cells and accelerate their release, such as macrophage and intestinal epithelial cell [15, 16]. Recently, supra-NPs shows great potential to combine the active and passive targeting strategies based on the advantages in its structure and function. Usually, β-cyclodextrin (β-CD), a critical component of supra-NPs, demonstrates various advantages such as easy availability, low cost, low toxicity and high biocompatibility [17]. More importantly, the cavity of β-CD can be bound with a variety of different guest molecules. By modifying the ligand on the guest molecules, the host-guest interaction can be taken advantage of to build the β-CD drug delivery system for the purpose of targeting. For example, to modify the specific ligands on the guest molecules, such as chondroitin sulfate (CS) [18, 19], hyaluronic acid (HA) [20] and D-Mannose (D-Man) [21], can help improve the uptake rate of supra-NPs by binding the relevant receptors to macrophages. In addition, given the unique physicochemical properties of inflammatory macrophages, such as the acidic pH environment in macrophages [22] or the accumulation of reactive oxygen species (ROS) [23], the accurate intracellular sensitive can be achieved by introducing such pH/ROS-sensitive materials as β-amino ester (PBAE) and thioglycolic anhydride into the supra-NPs [24, 25].

**Scheme 1:**
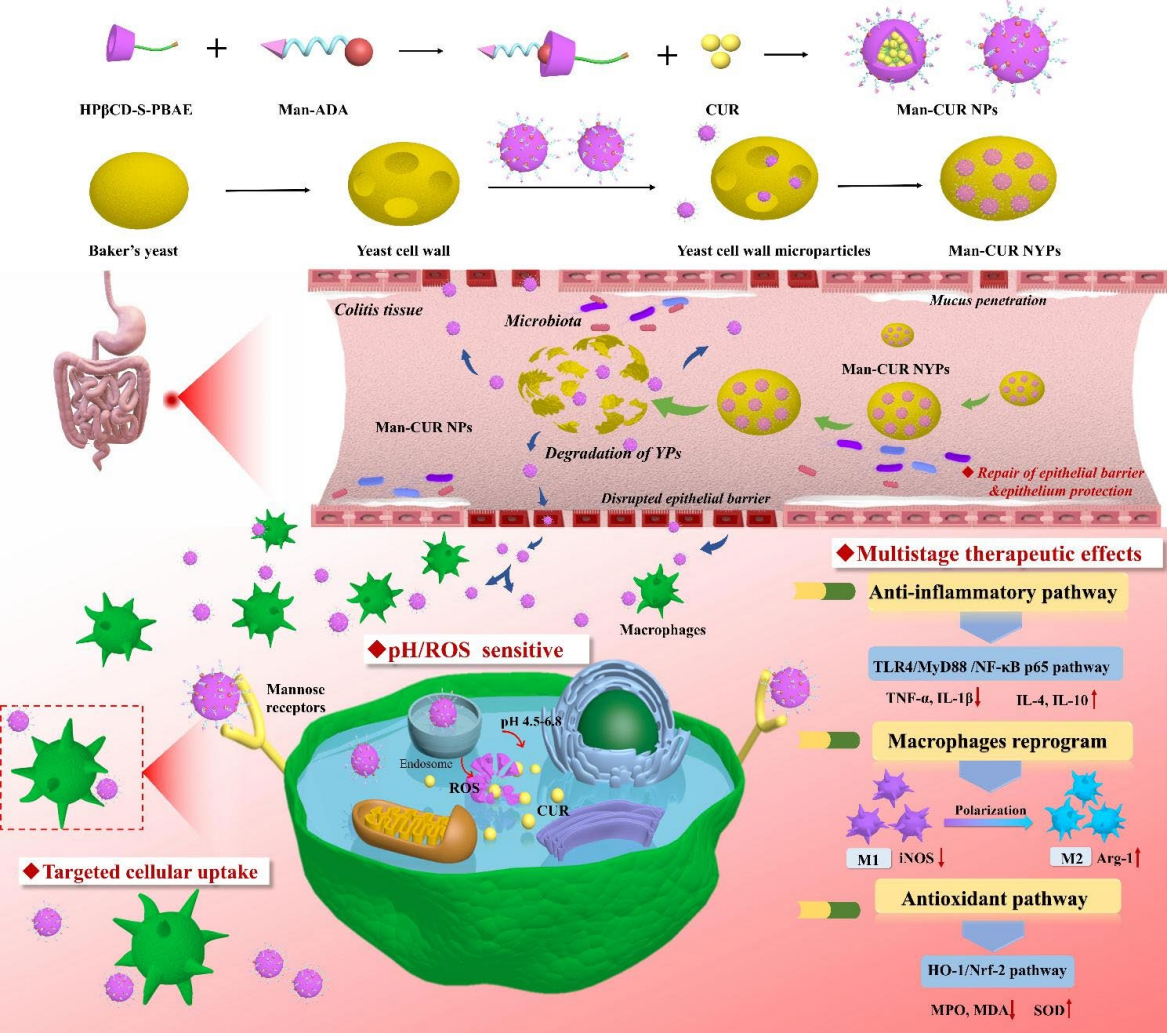
Fabrication process of Man-CUR NYPs and schematic illustration of treating UC through multistage therapeutic effects

Hence, in this study, supermolecule-based functional nanoparticles were combined with curcumin (CUR), a polyphenol compound with high therapeutic potency against UC (anti-inflammatory, anti-oxidant, macrophage reprogramming, etc.). We devised a “supramolecular curcumin nanoparticles with pH/ROS sensitive and multistage therapeutic effects” in “advanced yeast particles” delivery system (Scheme.1). Specifically, firstly, by taking advantage of the unique structure of β-CD, D-Man ligand is introduced to facilitate macrophage targeting. In addition, the intracellular sensitive releasing is achieved by taking advantage of the unique physicochemical properties of macrophages, the pH-sensitive PBAE and ROS-sensitive thioglycolic anhydride. Secondly, based on the drug-loading capability of YPs, YPs are used to encapsulate the Man-CUR NPs (Man-CUR NYPs), which can target delivery Man-CUR NPs to the inflammatory lesions and protect Man-CUR NPs against environmental assaults to improve the stability of Man-CUR NPs in oral delivery. To verify this hypothesis, the physicochemical properties and biomedical functions of Man-CUR NYPs were fully characterized *in vitro* and *in vivo*. By highlighting macrophage targeting and pH/ROS sensitivity of Man-CUR NPs, the gastric stability of Man-CUR NYPs is evaluated. Moreover, the anti-inflammatory effects of Man-CUR NYPs are examined through the TLR4/NF-κB signaling pathway, so as to evaluate macrophage polarization. Besides, the inhibitory effect on oxidation is analyzed through the Nrf2/HO-1 signaling pathway. To sum up, this study provides a practical reference for the application of YPs-encapsulated CUR supra-NPs in oral treatment of UC.

## Materials

CUR (≥ 98%, MW = 368.38) was purchased from Adamas-beta (Shanghai, China). Hydroxypropyl-beta-cyclodextrin (HPβCD) was purchased from Shangdong Binzhou Zhiyuan Biological Technology Co., Ltd (Shandong, China). D-Mannose (99%, MW = 180.16), 4-(dimethylamino)-pyridine (DMAP, 99%, MW = 122.17), 1-(3-Dimethylaminopropyl)-3-ethylcarbodiimide hydrochloride (EDC, 98%, MW = 191.7) and Thiodiglycolic anhydride (99%, MW = 132.13) were purchased from Macklin Biochemical Co., Ltd (Shanghai, China). 1,6-Hexanediol diacrylate (stabilized with MEHQ) (HDD, 99%, MW = 226.27), 4-Amino-1-butanol (AP, 98%, MW = 89.14), 1-Adamantanecarbonylchoride (97%, MW = 198.69) and 1-Hydroxybenzotriazole monohydrate (HOBT, ≥ 97%, MW = 135.13) were purchased from Aladdin Biochemical Technology Co., Ltd (Shanghai, China). Dimethyl sulfoxide was purchased from J&K Scientific Co., Ltd (DMSO, Beijing, China). The near-infrared lipophilic carbocyanine dye 1,1’-dioctadecyl-tetramethyl indotricarbocyanine iodide (DiR, > 95%, MW = 1013.39) and Dextran sodium sulfate (DSS, molecular weight: 36 − 50 kDa) were purchased from Dalian Meilun Biotechnology Co., Ltd (Dalian, China). 4′ ,6-Diamidino-2-phenylindole (DAPI) was obtained from Sigma-Aldrich Co. LLC.

The animal-related experiments gained approval from the Animal Ethics Committee of Chengdu University of Traditional Chinese Medicine and were arranged in accordance with the “Guidelines for the Care and Use of Laboratory Animals” by the Ministry of Science and Technology of China.

## Methods

### Copolymer synthesis and characterization

HPβCD-S-PBAE was prepared by means of esterification. Thiodiglycolic anhydride (3 mol) and EDC (4.5 mol) were dissolved in 20 mL DMSO to activate anhydride for 2 h. Then, HPβCD (1 mol), DMAP (1.5 mol) and HOBT (4.5 mol) were added for a 48-hour rection at 50℃. To remove DMSO and small molecular impurities, the resulting solution was dialyzed in deionized water with a dialysis bag (MWCO: 1200 Da). As the intermediate product, HPβCD-S was obtained by freeze-drying. Besides, PBAE was synthesized as described in our previous reports [19]. HPβCD-S (1 mol) and EDC (4.5 mol) were dissolved in 20 mL DMSO to activate anhydride for 2 h. Then PBAE (3 mol), DMAP (1.5 mol) and HOBT (4.5 mol) were added to react at 50℃ for 48 h. The obtained solution was dialyzed with deionized water in a dialysis bag (MWCO: 1200 Da) for 3 days. Through freeze-drying, the intermediate product of HPβCD-S-PBAE was obtained. In addition, D-Mannose was introduced into the HPβCD-S-PBAE complex via host-guest recognition for D-Mannose targeting. In summary, D-Mannose (1.5 mol) and 1-Adamantanecarbonylchoride (1 mol) were dissolved in 20 mL DMSO for an 18 h reaction at 60 ° C. Subsequently, the obtained solution was dialyzed in a dialysis bag (MWCO: 200 Da) with deionized water for 2 days, with the intermediate product obtained by freeze-drying (SCIENTZ-10 N, Ningbo Scientz Biotechnology Co., Ltd).

The chemical structure of PBAE, HPβCD-S and HPβCD-S-PBAE was characterized by proton nuclear magnetic resonance spectroscopy (1HNMR, AVANCE NEO 700 MHz spectrometer, Bruker, Germany). Besides, both HPβCD-S-PBAE and Man-AD were characterized by Fourier transform infrared spectroscopy spectroscopy (FTIR, Thermo Scientific Nicolet iS20 / Thermo Scientific Nicolet 6700, America). The number molecular weights and polydispersity index (PDI) of the synthesized polymers were determined by gel permeation chromatography (GPC, Agilent 1260 Infinity II, US) with DMSO as solvent.

### Preparation of NPs and NYPs

#### Preparation of Man-CUR NPs

Man-CUR NPs were prepared by using the nano-precipitation method. In brief, HPβCD-S-PBAE (30 mg) and Man-AD (4 mg) were separately dissolved in DMSO. Then, the resulting solution was added drop into water and sonicated for 15 min to obtain the carrier material. Next, 5 mg of CUR was dissolved in DMSO, and added dropwise into the mixed phase to form a nano-system. The solution was stirred at room temperature for 40 min, transferred to a dialysis bag (MWCO: 2000 Da) and dialyzed with deionized water to remove DMSO. In this way, Man-CUR NPs dispersion system was constructed. In addition, the free CUR was removed through 0.8 μm microporous membrane filtration. As a control group of Man-CUR NPs, non-targeted NPs were also prepared through simple nano-precipitation. The HPβCD-S-PBAE and CUR were dripped into the aqueous phase, and the subsequent operation was consistent with the above. In addition, unbonded PBAE nanoparticles were prepared (S-CUR NPs). The above process was conducted in strict light-proof conditions.

#### Preparation of Man-CUR NYPs

The yeast cell wall was extracted from baker’s yeast *Saccharomyces cerevisiae* as described in previous reports [11]. Man-CUR NYPs were obtained through the combination of electrostatic force-driven self-deposition and solvent hydration/lyophilization [8]. In brief, 150 mg of yeast cell wall was incubated with 15 mL of deionized water at 37 ° C for 30 min. Then, 15 mL of Man-CUR NPs solution was added and incubated for 1 h at room temperature. The obtained solution was centrifuged at 4000 rpm for 10 min, and the supernatant was replaced with deionized water. Subsequently, the suspension solution was subjected to a lyophilization − hydration cycle which was repeated twice. Meanwhile, water was used to force the nanoparticles loaded in pores or attached to the surfaces into the hollow core of YPs through a capillary action. Following the second-time hydration, Man-CUR NYPs were rinsed with deionized water to remove free NPs, followed by final lyophilization.

### Characterization of NPs and NYPs

#### Characterization of Man-CUR NPs

The average hydrodynamic particle size (nm) and zeta potential (mV) of CUR NPs, and Man-CUR NPs were measured by means of dynamic light scattering (DLS) with a Particle Analyzer Lite sizer 500 (Anton Paar, Austria). The morphology of Man-CUR NPs was examined using transmission electron microscope (TEM, JEM 1200X, JEOL, Japan). In addition, the X-ray powder diffraction (XRD) spectra of CUR, Man-CUR NPs, Blank NPs and mixture of CUR and all carrier matrix were recorded by XRD diffractometer (Rigaku Smartlab, Japen) from 5 ° to 90 ° at a scanning speed of 5 °/min .

The encapsulation efficiency (EE, %) and loading efficiency (LE, %) of Man-CUR NPs were determined through the high-performance liquid chromatography (HPLC, LC-45202-46, Shimadzu, Kyoto, Japan) method. Prior to HPLC analysis, NPs were dissolved in methanol to destroy the structure of NPs and release CUR. The formula used to calculate EE and LE is as follows: EE (%) = amt of CUR loaded/amt of CUR added × 100%; LE (%) = amt of CUR loaded/total amt of NPs harvested × 100%.

#### Characterization of Man-CUR NYPs

The average hydrodynamic particle size of YPs and Man-CUR NYPs was measured by using a Laser particle sizer (Malvern Mastersizer 2000). The morphology of YPs and Man-CUR NYPs was examined by scanning electron microscope (SEM, ZEISS Sigma 300, Germany). YPs were labeled with calcofluor white (fluorescent dye). The formation of NYPs and the loading of NPs on YPs were observed under confocal laser scanning microscopy (CLSM, TCS SP8 SR, Leica, Weztlar, Germany). In addition, three-dimensional confocal imaging technique was used to observe the truncated Man-CUR NYPs images. To investigate whether Man-CUR NPs are located in the core of YPs.

### Stability evaluation of NYPs

In order to evaluate the stability of Man CUR NYPs in simulated gastric juice (SGF) and simulated colonic fluid (SCF), the methods as mentioned previously were adopted. CUR NYPs were immersed in 9 mL of SGF (pH 2, containing 1 mg/mL pepsin) and 9 mL SCF (pH 7.4, containing 0.5% β-glucanase) for the 4 h oscillation in a shaker at 100 rpm at 37 ° C [12]. The morphology of NYPs was examined by SEM.

### pH/ROS sensitivity of Man-CUR NPs

The pH value and oxidation properties of Man-CUR NPs were studied under different conditions. The oscillation of Man-CUR NPs was performed at 100 rpm, 37 ° C for 4 h in the phosphate buffered normal saline (PBS) with a pH value of 6.0 or containing 1 mM H_2_O_2_ with a pH value of 7.4, respectively [26, 27]. After incubation, the average particle size of Man-CUR NPs was measured by DLS and their morphology was examined by SEM.

### *In vitro* drug release profile

The *in vitro* release characteristics of CUR as prepared under different conditions were studied by using the dialysis method. In briefly, 2 mL of Free CUR, Man-CUR NPs and Man-CUR NYPs (at a concentration of 100 µg/mL) were added separately into the dialysis bag (MWCO: 3000 Da). Meanwhile, tween 80 was added into the system, the mass concentration of which was adjusted to 0.5% (w/v) for the improved solubility of CUR in the release medium, so as to facilitate content determination. In addition, 0.5% β-glucanase (pH 7.4) was added to a release medium to evaluate the release behavior of Man-CUR NYPs. Then, the dialysis bag was immersed in 30 mL of different release media and placed in a shaker at 37 ℃ for 100 rpm. With 1 mL of samples taken out at each preset time point, 1 mL of new release medium was added, with the cumulative release determined by HPLC.

### Intracellular uptake of NPs

In order to assess the cellular uptake efficiency of different ways of CUR preparations, the qualitative analysis of cellular uptake was conducted by Confocal laser scanning microscopy (CLSM; TCS SP8 SR; Leica, Weztlar, Germany). RAW264.7 macrophages were incubated with Free-CUR, CUR-NPs and Man-CUR NPs (CUR content of 32 µg/mL) for 4 h, respectively. In the meantime, the D-Man preincubated RAW264.7 macrophages were incubated with CUR-NPs and Man-CUR-NPs for 4 h. Then, the cells were stained with 4′ ,6-Diamidino-2-phenylindole (DAPI) and the uptake was examined under CLSM. In addition, flow cytometry (FCM; NovoCyte; ACEA, San Diego, CA, U.S.A.) was performed for the quantitative analysis of cellular uptake. For time-dependent study, Man-CUR NPs were incubated with cells for different periods of time (0.25, 0.5, 1, 2 and 4 h). To carry out concentration-dependent study, the Man-CUR NPs of different concentrations (CUR concentrations of 2,4,8,16, and 32 µg/mL) were incubated with cells for 4 h. To ascertain the variations in cellular uptake between different CUR-loaded preparations, the cells were separately co-incubated with Free-CUR, CUR-NPs, and Man-CUR NPs (32 µg/mL CUR) for 4 h. To investigate the targeting of D-Man on macrophage Mannose receptor, the serum-free medium containing D-Man (10 mg/mL) was pre-incubated with the cells for 1 h. Then, it was incubated with CUR-NPs and Man-CUR NPs for 4 h. Finally, FCM quantitative analysis was conducted on cell uptake.

### *In vitro* anti-inflammatory, antioxidant activities and macrophage polarization

To explore the anti-inflammatory, antioxidant and Macrophage polarization effects of Man-CUR NPs *in vitro*, LPS-induced RAW264.7 cells were used as the cell model [28]. In brief, RAW264.7 macrophages were inoculated in 12-well plates and cultured overnight. Then, they were co-cultured for 12 h through different CUR-loaded preparations (Free-CUR, CUR-NPs and Man-CUR NPs, of which CUR content is 16 µg/mL). Next, the obtained cells were washed with PBS and treated with LPS (1 µg/mL) for 3 h [29, 30]. Finally, the level of such inflammatory (TNF-α, IL-1β), anti-inflammatory (IL-4, Il-10), and oxidation (MDA, SOD) cytokines in the supernatant was examined by the corresponding enzyme-linked immunosorbent assay (ELISA) kits according to manufacturer’s instructions (Multi Sciences (Lianke) Biotech, CO., LTD, Hangzhou, China). In addition, Dihydroethidium (DHE) fluorescent probe was used to incubate LPS-induced cells for 20 min at room temperature. Then, the cells were fixed in 4% paraformaldehyde, and the nuclei were stained with DAPI. Finally, the macrophages were photographed by CLSM. In this experiment, untreated cells were taken as the negative control and LPS-stimulated cells were treated as the positive control. In order to explore the ability of Man-CUR NPs to transform macrophages into M2 phenotype, immunofluorescence (IF) and flow cytometry analysis was performed. CD206 and Arg-1 were applied as primary antibodies, coralite 594 was treated as secondary antibodies and the nucleus was dyed with DAPI. The expression level of the protein was measured by CLSM.

To further verify the polarization effect of nanoparticles on macrophages. RAW264.7 macrophages were stimulated with 1 µg/mL LPS to transform into M1 type and 20 ng/mL IL-4 to transform into M2 type. The morphological changes of RAW264.7 macrophages were observed by microscope. Different CUR-loaded preparations (Free-CUR, CUR-NPs and Man-CUR NPs, of which CUR content is 16 µg/mL) were subsequently co-incubated with LPS-induced macrophages. Finally, the expression levels of M1 macrophage marker CD86 and M2 macrophage marker CD206 were detected by flow cytometry.

### *In vivo* biodistribution evaluation

To localization and biodistribution of different ways of preparation were evaluated after the oral administration for UC model mice. In brief, 3% DSS was used to induce UC mice models, and different DiR preparations were made by using DiR instead of CUR. Then, mice were randomly divided into 4 groups and treated with DiR-loaded preparations (Free-DiR, Man-DiR NPs, DiR NYPs and Man-DiR NYPs) at the same concentration (3 mg/kg) [31]. Then, the Caliper Life Sciences LIVIS® Lumina Series (PerkinElmer, Waltham, MA, USA) was employed to take photos at a preset interval (3, 6, 12 and 24 h). Finally, the mice were sacrificed 24 h, with the colon collected and photographed. Also, the localized fluorescence intensity was analyzed with *in vivo* imaging software.

### *In vivo* target properties

The targeted macrophage of nanoparticles *in vivo* was studied. The oral gavage of Free CUR, Man-CUR NPs, CUR NYPs and Man-CUR NYPs was performed for UC mice, with the dosage of CUR set to 5 mg/kg. After 12 h of administration, the mice were sacrificed and the colon was collected. Then, cut the embedded colon tissue into a slice of 20 μm thick (Cryostat Microtome, Leica CM1950, Nussluch, Germany). After repairing and blocking, the slices were incubated with F4/80 polyclonal antibody at 4℃ overnight, and then stained with Coralite594; and the nucleus were stained with DAPI. Finally, the fluorescence of the tissue was observed under Fluorescence microscope.

### *In vivo* therapeutic effect

The BALB/c mice were fed with DSS (3%, w/V) for an 8-day periods to establish the UC model. They were randomly divided into 8 groups (*n* = 6): (1) Normal, (2) Model, (3) Free CUR, (4) S-CUR NPs, (5) CUR NPs, (6) Man-CUR NPs, (7) CUR NYPs, and (8) Man-CUR NYPs. After adaptive feeding, they were fed with 3% DSS for 3 days except for the normal group. The mice were treated with 5 mg/kg CUR from day 4 to day 10, while the normal group was allowed normal saline only. Throughout the experiment the changes in body weight, visible stool consistency, fecal bleeding and disease activity index (DAI) were all recorded. The mice were sacrificed on the last day of the experiment, with colons and major organs collected. The length of the colon was measured and the spleen was weighed. The obtained colon tissues were fixed with 10% paraformaldehyde, immersed in paraffin, cut into the slices sized 5 μm and stained separately with hematoxylin eosin (H&E) and periodic acid-Schiff (PAS). Besides, the heart, liver, spleen, lungs and kidneys of each mouse were collected and stained with H&E for histopathological analysis. Whole serum were collected for biochemical tests. Then, the morphological changes of colon tissue were observed under light microscope, to evaluate the severity of inflammation, crypt damage and ulcer. The expression level of TNF-α, IL-1β, IL-4 and IL-10, MPO, SOD, and MDA in colon tissues was measured with the assistance of commercially available ELISA kits. Western blotting was performed to examine the effects on anti-inflammatory (TLR4/ MyD88/ NF-κB p65) and antioxidant signaling pathways (Nrf-2/ HO-1) as well as the expression of M1 and M2 macrophage-associated proteins (iNOS/ Arg-1). With β-actin antibodies as the internal control, the equal loading of protein was confirmed. The captured chemiluminescence signals were analyzed using ImageJ software.

### Immunohistochemistry assay of the colon tissues

To investigate the effect of Man-CUR NYPs on macrophage polarization, the expression of CD86, iNOS, CD206 and Arginase-1(Arg-1) in the colon of mice was determined by immunohistochemistry (IHC). In order to reveal the effect of Man-CUR NPs on the expression of colonic tight junction proteins, IHC was also adopted to determine the expression of ZO-1, Claudin-1 and Occludin in the colon of mice.

### Statistical analysis

All data were expressed as mean ± SD. Statistical analysis was carried out the form of one-way ANOVA test, with a *p* value < 0.05 treated as significant.

## Results and discussion

### Analysis of synthetic HPβCD-S-PBAE/copolymer synthesis and characterization

**Fig. 1:**
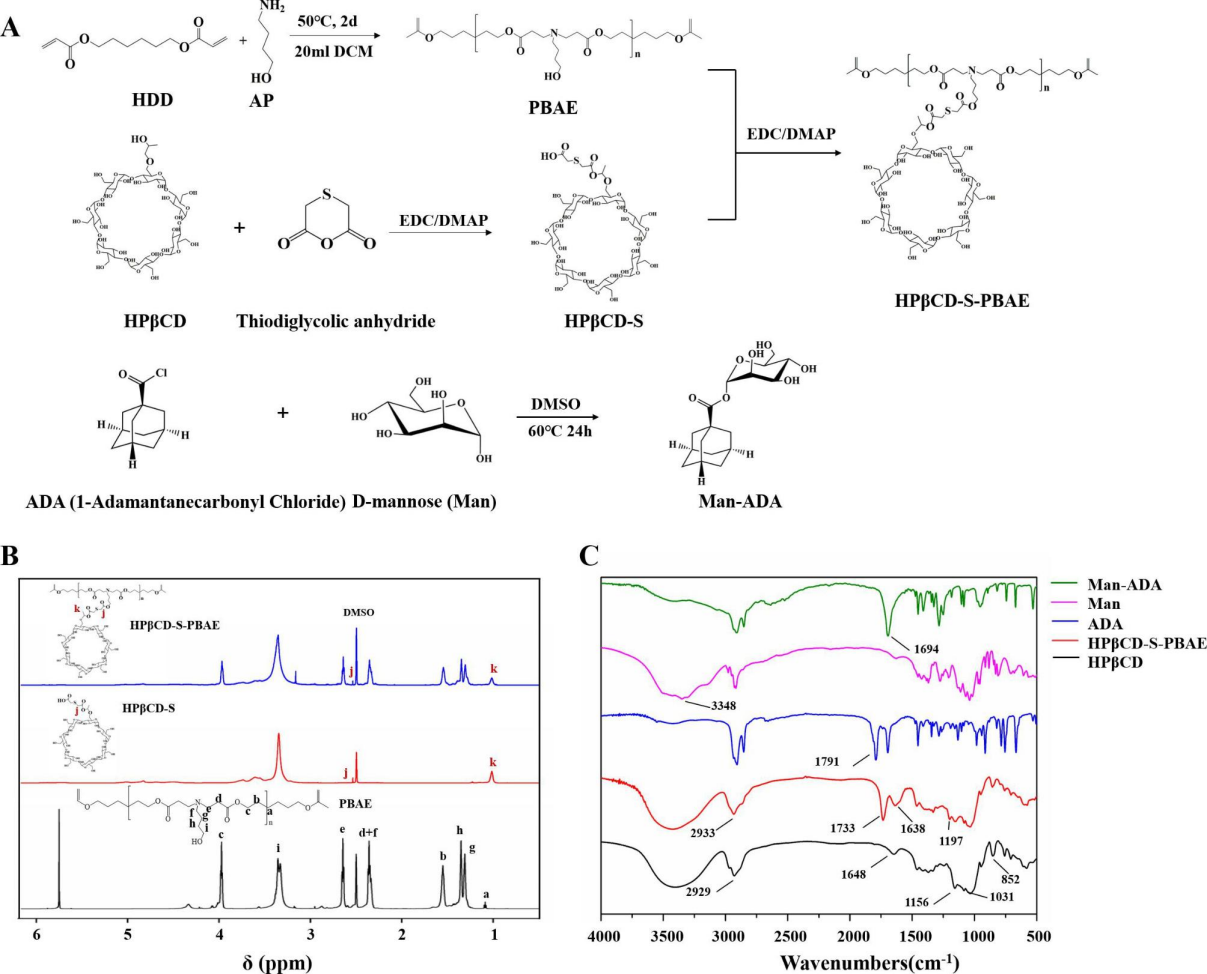
Copolymer synthesis and characterization: (**A**) Synthesis route of HPβCD-S-PBAE and Man-ADA. (**B**) 1 H NMR spectra of HPβCD-S-PBAE. (**C**) FTIR spectra of HPβCD-S-PBAE and Man-ADA.

Herein, a novel pH/ROS dual responsive polymer carrier (HPβCD-S-PBAE) was obtained through the synthesis as enabled by grafting thioglycolic anhydride onto HPβCD and then grafting PBAE at the strong hydrophobic end. Meanwhile, a nano-system was developed for the purpose of drug sensitive release. The synthesis of HPβCD-S-PBAE is shown in Fig. 1A. Specifically, PBAE is synthesized by means of Mikell addition polymerization and HPβCD-S-PBAE is synthesized through esterification. Figure 1B shows the ^1^ H NMR spectra of PBAE, HPβCD-S and HPβCD-S-PBAE, respectively. Retained in the ^1^ H NMR spectrum of PBAE, the original proton peaks of − NCH_2_CH_2_CH_2_CH_2_OH of AP are located at 2.34 (f), 1.30 (g), 1.34 (h) and 3.34 (i), respectively. The peaks of 1.08 (a), 1.54 (b), 3.96 (c) and 2.34 (d) are ascribed to the HDD unit [19]. In the ^1^ H NMR spectrum of the HPβCD-S and HPβCD-S-PBAE polymer, a characteristic peak emerges at 2.53 (j), which results from COCH2SCH2COO- and -OOCCH_2_SCH_2_COO-, respectively. According to the ^1^ H NMR spectra of HPβCD-S-PBAE polymer, the characteristic peaks resulting from PBAE are retained. In addition, HPβCD-S and HPβCD-S-PBAE also retain the proton peak of hydroxypropyl-CH_3_ on the original HPβCD, the location of which is at 1.01(k). Figure 1 C shows the FTIR of the polymers. The spectrum of HPβCD indicates a broad band between 3700 and 3050 cm^− 1^ and a band in the 2930 cm^− 1^ region, which characterizes a C-H stretch proper to sugars. The strong peak at 1733 cm ^− 1^ is ascribed to the stretching vibration of carbonyl C = O on the HPβCD-S-PBAE ester bond, while the strong peak at 1193 cm^− 1^ results from the bending vibration of C-N single bond on the main chain of HPβCD-S-PBAE. In addition, the strong peak at 1791 cm ^− 1^ is ascribed to C-CO-Cl telescopic vibration, while the strong peak at 1694 cm^− 1^ results from the stretching vibration of carbonyl C = O on the Man-ADA ester bond. It implies the successful synthesis of Man-ADA. The GPC analysis results (Table 1) showed that the Mz of P HPβCD-S-PBAE is approximately 5325 for the following experiments. All the aforementioned results confirmed that HPβCD-S-PBAE was successfully synthesized.

**Table 1:** GPC data of HPβCD-S-PBAE and its precursors

Samples	Mz (g/mol)	PDI
HPβCD-S	3166	1.13
PBAE	2352	1.09
HPβCD-S-PBAE	5325	1.55

### Characterization of NPs and NYPs

**Fig. 2:**
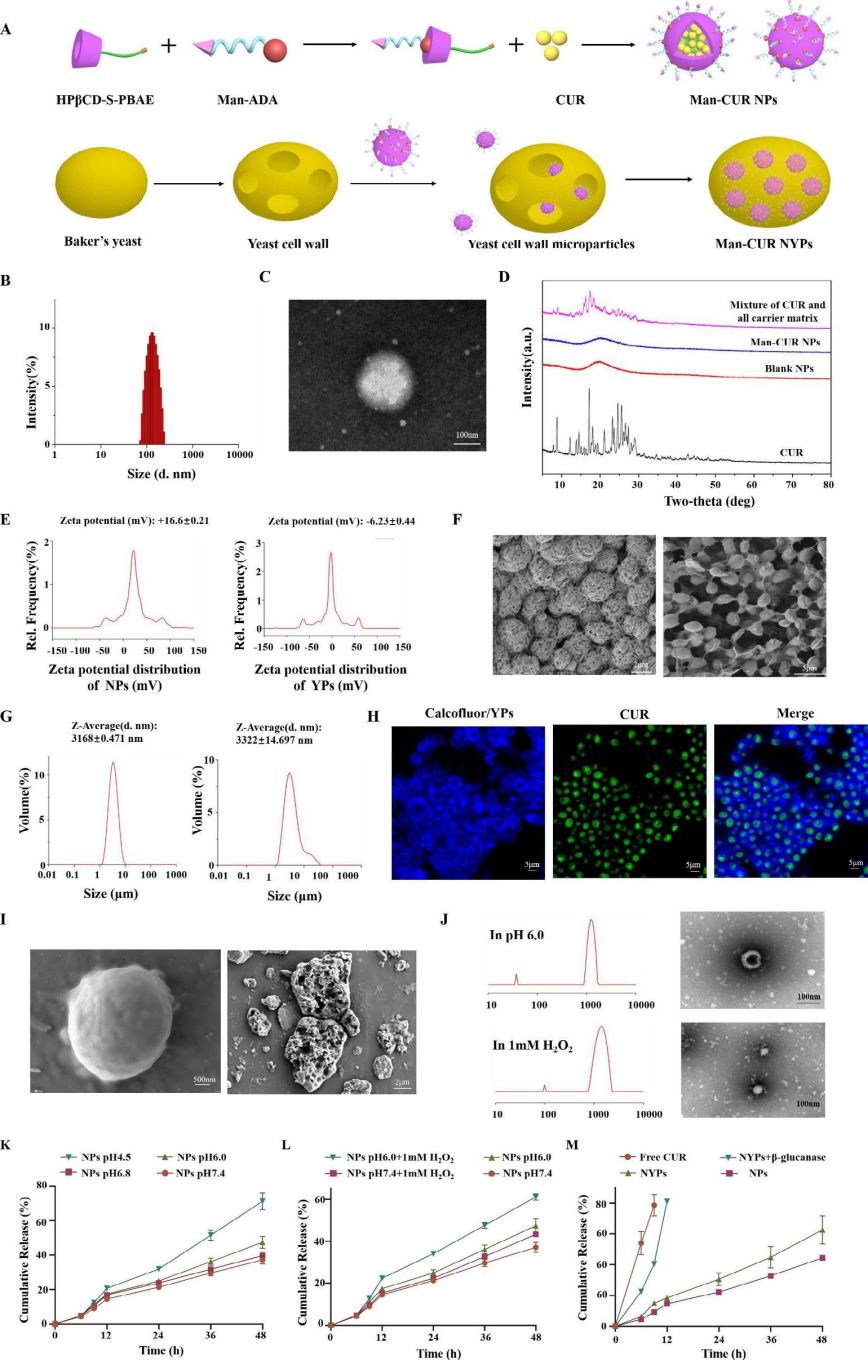
Characterization, stability, and release behavior of Man-CUR NPs and Man-CUR NYPs: (**A**) Fabrication process of Man-CUR NPs and Man-CUR NYPs. (**B**) Size distribution of Man-CUR NPs. (**C**) TEM image of Man-CUR NPs. (**D**) XRD analysis. (**E**) Zeta potential distribution of NPs and YPs. (**F**) SEM image of YPs and Man-CUR NYPs. (**G**) Size distribution of YPs and Man-CUR NYPs. (**H**) Representative CLSM images of Man-CUR NPs (green) and the yeast cell wall was labeled with calcofluor white (blue; scale bar = 25 μm). (**I**) Stability evaluation of NYPs. (**J-M**) The size distribution, TEM and cumulative release profiles of CUR under different conditions. Data are shown as means ± SD (*n* = 3)

The preparation of CUR-loaded oral drug delivery systems involves two steps, as shown in Fig. 2A. One is the preparation of Man-CUR NPs and the other is to prepare Man-CUR NYPs by loading Man-CUR NPs into YPs. Therefore, the characterization section includes Man-CUR NPs and Man-CUR NYPs.

### Characterization of Man-CUR NPs

Man-CUR NPs were prepared by means of nano-precipitation method and characterized through a combination of size distribution, zeta potential, EE, LE, TEM and XRD. HPβCD-S-PBAE was synthesized through Michael addition and esterification, and CUR was loaded with this complex. According to the results, the entrapment is highly efficient, with the EE and LE are about 90.24%±1.49% and 8.54 ± 0.08%. The particle size of Man-CUR NPs is nearly 143.44 ± 2.05 nm (Fig. 2B), and the zeta potential is about + 16.6 ± 0.21 mv. TEM images were produced to confirm the uniform spherical structure of Man-CUR NPs (Fig. 2C). To sum up, Man-CUR NPs are capable of controllable drug loading and have a particle size suitable for the delivery of CUR. In addition, the join of PBAE plays a significant role in the loading of NPs. Loading CUR with HPβCD alone can lead to very low efficiency of encapsulation, as shown in the Figure S1.

Given a vital role played by the molecular interaction between the drug and the carrier in the characteristics of drug release, the corresponding XRD spectra were studied. As shown in Fig. 2D, there are plenty of sharp peaks in the XRD pattern of free CUR in the range of 5 ° ~ 90 °, indicating a highly crystalline state. However, these peaks are not observable in Man-CUR NPs, indicating the absence of crystal complex between CUR and HPβCD-S-PBAE matrix. Therefore, CUR doses exist in amorphous or disordered crystal state.

According to the above results, Man-CUR NPs are the spherical nanoparticles with uniform particle size and high entrapment efficiency. CUR exists in Man-CUR NPs in the form of amorphous or disordered crystals.

### Characterization of Man-CUR NYPs

Man-CUR NYPs were prepared by the method of “electrostatic adsorption-vacuum extrusion-electrostatic distribution-hydration rearrangement” [8, 32]. Due to the residual carboxyl groups present on the surface of yeast cell wall, blank YPs showed a negative potential of -6.23 ± 0.44 mV (Fig. 2E). Therefore, the positively charged NPs are drawn by electrostatic adsorption into YPs cavity or adhere to its surface. By repeating the freeze-drying-hydration process, two results are produced. On the one hand, the captured SEM images of Man-CUR NYPs indicate the disappearance of voids from the surface compared with blank YPs, showing a smooth surface morphology, and spherical particles are observable (Fig. 2F). On the other hand, the mean hydrodynamic diameter of Man-CUR NYPs is 3322 ± 14.697 nm after loading, which is insignificantly different than blank YPs (3168 ± 0.471 nm) (Fig. 2G). It suggests that NPs exist in the core of YPs, not on the surface of YPs. The above results show that Man-CUR NPs migrates into YPs through pores, thus filling its cavity to form a smooth spherical structure.

In order to confirm the hollow structure of empty YPs and intuitively verify whether Man-CUR NPs are loaded in YPs directly, the fluorescent dye calcofluor white selectivity bound to glucan wall was used for CLSM imaging. As shown in Fig. 2H, a hollow structure is observable in YPs, which is consistent with the report [33]. More importantly, Man-CUR NPs (green fluorescence) infiltrate the core of YPs, indicating the successful and efficient loading of Man-CUR NPs into YPs. Furthermore, 3D images (Figure S2) further indicate that Man-CUR NPs are located at the core of the YPs and that Man-CUR NPs fill the entire core, achieving a payload for Man-CUR NPs. It is noteworthy that this study is the first to propose the encapsulation of drug-loading NPs into YPs directly, not the probe labeled NPs published by us previously [12, 13].

### Stability evaluation of NYPs

In order to establish whether YPs can protect NPs from the harsh gastric environment and release NPs in the colon, Man-CUR NYPs were evaluated for their stability in SGF and SCF. Figure 2I shows the SEM images of Man-CUR NYPs as captured after 4 h of incubation in SGF solution. These images show the relatively complete morphology, which is similar to untreated Man-CUR NYPs, indicating the stable existence of Man-CUR NYPs in SGF solution. Notably, both incomplete morphology and cracking can be observed from the SEM images of Man-CUR NYPs as captured after the incubation in SCF solution. According to these results, Man-CUR NYPs are highly effective in offering protection in the gastrointestinal environment, and capable to release nanoparticles or drugs in the colon. Therefore, YPs are promising for clinical use as an oral colon delivery carrier of drugs or NPs.

### pH-/ROS- single response capability of Man-CUR NPs

In order to verify the single sensitivity of Man-CUR NPs to pH or ROS, DLS and TEM were applied separately to measure the particle size distribution and morphological changes of Man-CUR NPs exposed to different simulated environments for 4 h. As shown in Fig. 2J, the particle size of Man-CUR NPs increases after 4 h of incubation given a pH value of 6.0 or 1 mM H_2_O_2_, even with heterogenous peaks appearing. As confirmed by TEM results, acidic environment or H_2_O_2_ has effects on Man-CUR NPs, thus resulting in the irregularity of NPs surface. It is suspected that pH sensitivity may be attributable to PBAE protonation, and that ROS sensitivity results from the breakdown of thioglycolic anhydride in NPs after the reaction with H_2_O_2_. These results are the potential evidence of single sensitivity shown by Man-CUR NPs in the presence of reactive oxygen or under acidic conditions. However, a further study is required to verify the dual sensitivity of Man-CUR NPs for the evaluation of release behavior.

### *In vitro* release evaluation

Firstly, the drug release behavior of Man-CUR NPs in buffer was studied at different pH values (7.4, 6.8, 6.0 and 4.5). These buffers with different pH values were used to simulate the endosome (pH 6.0-6.8) and mature lysosome (pH 4.5) respectively [27]. As shown in Fig. 2K, approximately 37% of the loaded CUR was released after the NPs were incubated in the buffer with a pH of 7.4 for 48 h. With the reduction in pH value, the drug release rate increases significantly. Especially in the buffer solution with a pH of 4.5, NPs show a higher drug release rate than in other buffer solutions, with a cumulative release of 71.20% achieved after 48 h. These results demonstrated that the release rate is improved under acidic environments, which is possibly attributed to the breaking of ester bond in PBAE.

In addition, an examination was conducted on the synergistic effect between pH and ROS stimulators to simulate the drug release behavior in the macrophage environment in the colitis site. The controlled release curves of drugs are included under the conditions of pH 7.4 buffer, pH 7.4 buffer containing 1 mM H_2_O_2_, pH 6.0 buffer, and pH 6.0 buffer containing 1 mM H_2_O_2_ (Fig. 2L). It was found out that the drug release rate was significantly increased in the buffer containing H_2_O_2_. At the pH value of 6.0, only about 47% of the encapsulated drug was released within 48 h. However, when 1 mM H_2_O_2_ was added to the buffer with a pH of 6.0, the cumulative drug release improved to about 61%, indicating the ROS-responsive capacity of Man-CUR NPs. This burst may be attributable to the oxidation of thioglycolic anhydride. The results are a reduction in the structural stability of NPs, which promotes the release of encapsulated drugs. Furthermore, through a further comparison between pH 7.4, and pH 6.0 + H_2_O_2_, it was discovered that the release increased from approximately 37–61%, which illustrates the dual pH/ROS responsiveness of Man-CUR NPs.

An analysis was also conducted on the release of Free CUR, Man-CUR NPs, Man-CUR NYPs and Man-CUR NYPs containing 0.5% β-glucanase in the buffer with a pH of 7.4. As shown in Fig. 2M, the release rate of the Man-CUR NPs and Man-CUR NYPs is significantly lower compared to Free CUR. Notably, the release of CUR from Man-CUR NYPs becomes faster in the presence of 0.5% β-glucanase, indicating that NYPs are capable of lysis in the colonic environment. To sum up, Man-CUR NYPs can enable the targeted release in the presence of β-glucanase produced by intestinal flora.

According to the results show, Man-CUR NYPs can release Man CUR NPs under the action of intestinal flora. Then, Man-CUR NPs induce a pH/ROS dual response within macrophages to release drugs. As a result, controlled drug release is achieved through the synergistic effect of internal pH/ROS stimulation.

### *In vitro* cell internalization profiles of NPs

**Fig. 3:**
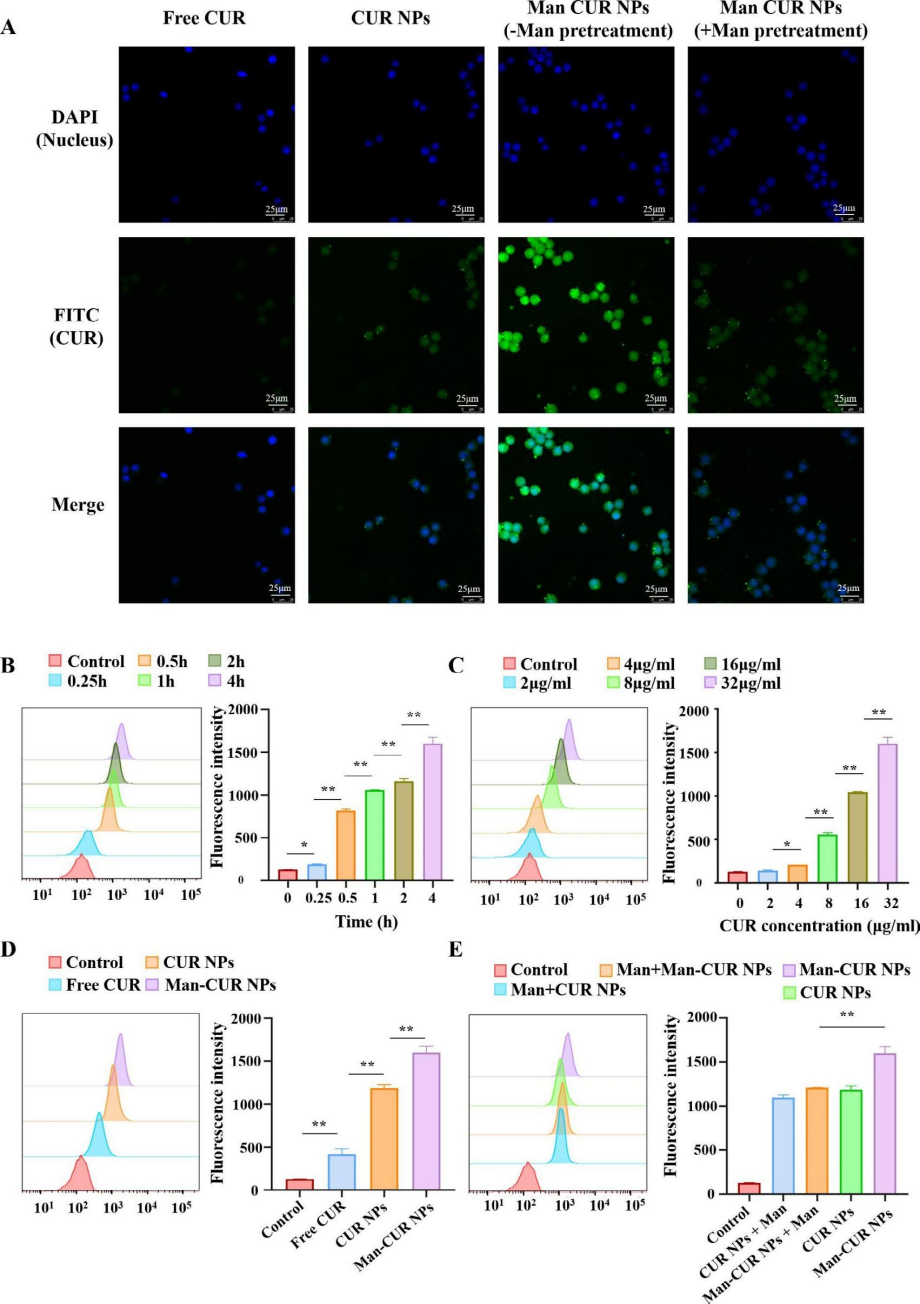
Cell internalization profiles of NPs: (**A**) Qualitative analysis of uptake by RAW264.7 macrophages: (**B**) Quantitative measurement of CS-CUR NPs uptake by RAW264.7 macrophages (incubation times of 0, 0.25, 0.5, 1, 2, and 4 h; CUR concentration of 32 µg/mL). (**C**) Quantitative test of Man-CUR NPs uptake by RAW264.7 macrophages (4 h, different CUR concentrations 2, 4, 8, 16, and 32 µg/mL). (**D**) Quantitative uptake measurement by RAW264.7 macrophages treated with different formulations (4 h, CUR concentration of 32 µg/mL). (**E**) Quantitative result uptake by RAW264.7 macrophages incubated with CUR NPs and Man-CUR NPs with or without the pretreatment of D-Man. Data are shown as mean ± SD (*n* = 3). ⁎*p* < 0.05, ⁎⁎*p* < 0.01

YPs can protect Man-CUR NPs effectively through the upper gastrointestinal tract, and then release the Man-CUR NPs in the colon. Therefore, in order to simulate the process of drug uptake *in vivo*, the cell internalization of Man-CUR NPs was studied. Both CLSM and FCM were applied to qualitatively and quantitatively analyze the uptake of NPs by RAW264.7 macrophages. For the CLSM study, as shown in Fig. 3A, compared with Free CUR, the macrophages incubated with NPs for 4 h show a higher level of green fluorescence. Moreover, the macrophages incubated with Man-CUR NPs observed stronger fluorescent signals than those incubated with CUR NPs, suggesting the role of D-Man in targeting macrophages and its greater capacity to promote NPs internalization in cells. In addition, to assess whether Man-modified NPs could improve the targeted cellular uptake of macrophages by Mannose receptor. The macrophages were pretreated with D-Man for 1 h. It was observed that the green fluorescence of Man-CUR NPs was significantly weakened, indicating a significant decrease in cellular uptake efficiency. It is suggested that Man-CUR NPs can be specifically internalized into macrophages by Mannose receptor.

In order to further explore the internalization efficiency, FCM was applied to quantitatively analyze the efficiency of cell uptake. As shown in Fig. 3B, the fluorescence intensity of Man-CUR NPs increases over time from 0 to 4 h, indicating that uptake of Man-CUR NPs is time-dependent in RAW264.7 macrophages. Meanwhile, the fluorescence intensity of Man-CUR NPs rises with the increase in dose from 0 to 32 µg/mL, indicating a dose-dependent cellular uptake in RAW264.7 macrophages (Fig. 3C). After the incubation of RAW264.7 macrophages with Free CUR, CUR NPs and Man-CUR NPs at a concentration of 32 µg/mL for 4 h, the fluorescence intensity of Free CUR was the weakest (Fig. 3D), indicating that nano-CUR is more rapidly absorbed by the cells. In addition, since Man has a targeting effect of mannose receptor, the fluorescence intensity of Man-CUR NPs is significantly higher compared to CUR NPs (*p* < 0.01). In addition, compared to the Man-CUR NPs without Man pretreatment, the fluorescence intensity of Man-CUR NPs is significantly reduced after Man pretreatment (Fig. 3E; *p* < 0.01), which is basically consistent with the results of CLSM. To sum up, it is demonstrated that the specific binding of Man to Mannose receptor is effective in promoting the uptake of NPs by macrophages. Furthermore, it was verified that the Man in the NPs could target macrophages.

### *In vitro* anti-inflammatory, antioxidant activities and macrophage polarization

**Fig. 4:**
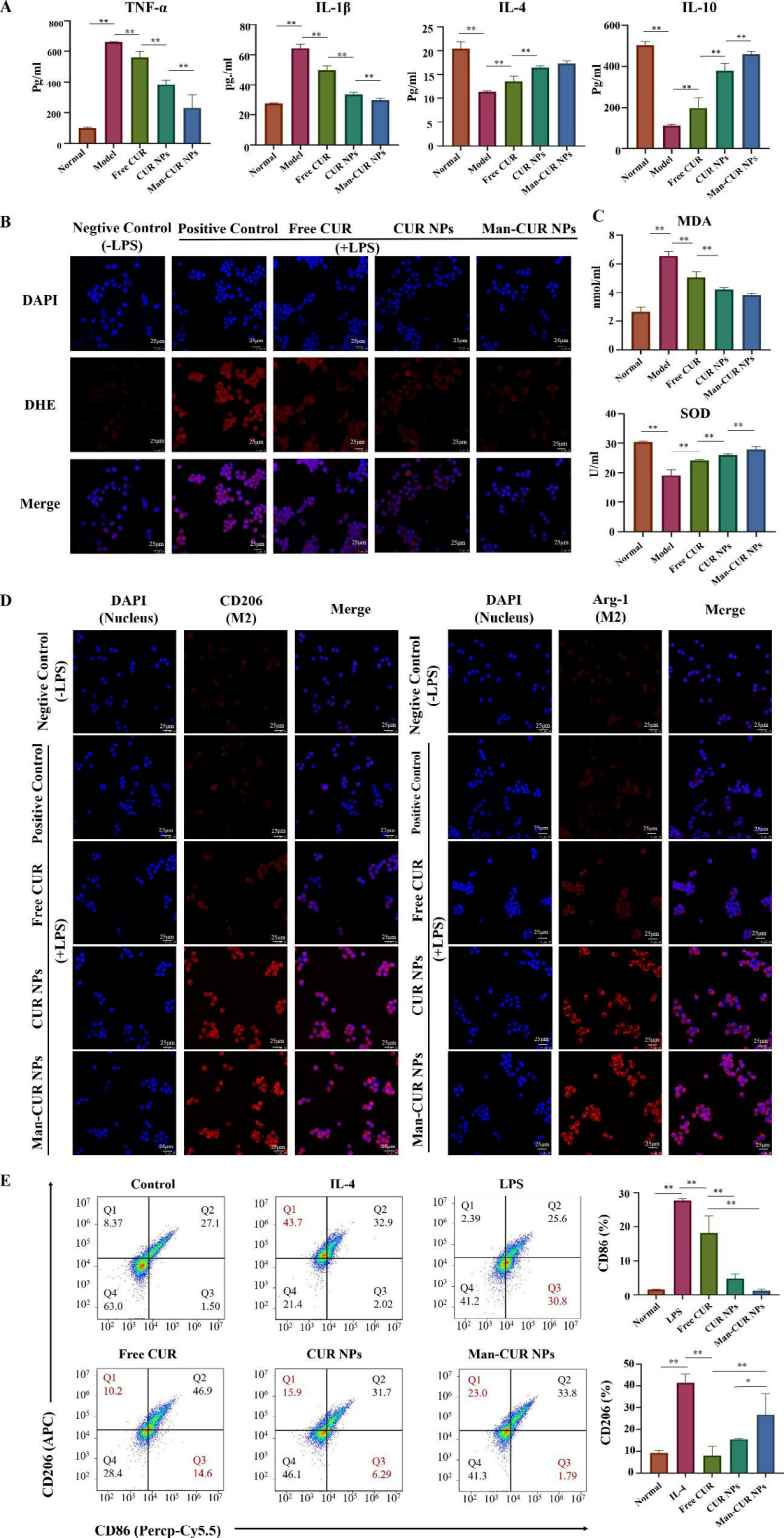
*In vitro* anti-inflammatory activities and antioxidant properties of NPs. Comparative studies of *in vitro* anti-inflammatory activities among different formulations (CUR concentration of 10 µg/mL) in terms of (**A**) TNF-α, IL-1β, IL-4, IL-10. (**B**) In vitro antioxidant activities of RAW264.7 macrophages after treatment with different formulations (CUR concentration of 10 µg/mL) for 12 h. Scale bar = 25 μm. (**C**) CD206 and Arg-1 in peritoneal macrophages after treatment with different formulations (CUR concentration of 10 µg/mL) for 12 h. Scale bar = 25 μm. (**D**) Comparative studies of in vitro antioxidant activities among different formulations (CUR concentration of 10 µg/mL) in terms of MPO and SOD. (**E**) The percentage of CD86 and CD206 were detected by flow cytometry. Data are mean ± SD (*n* = 6). *p < 0.05; **p < 0.01

The *in vitro* pharmacologic research of Man-CUR NPs was conducted by using LPS-induced RAW264.7 macrophages, as showed in Fig. 4. According to Fig. 4A, compared with the negative control cells, LPS-treated (positive control) cells significantly increase the secretion of pro-inflammatory cytokines TNF-α and IL-1β (*p* < 0.01). Compared with the positive control cells, CUR NPs and Man-CUR NPs significantly reduced the secretion of TNF-α and IL-1β (*p* < 0.01), indicating the anti-inflammatory activity. In addition, the cells treated with Man-CUR NPs secreted significantly lower concentrations of pro-inflammatory cytokines than the cells treated with CUR NPs. These results show that Man-CUR NPs can inhibit the secretion of inflammation through activated macrophages. In addition, the anti-inflammatory cytokines IL-4 and IL-10 exhibit the opposite trend to TNF-α and IL-1β.

The production and accumulation of excessive reactive oxygen species (ROS) can induce cell damage and oxidative stress [34]. As a variety of natural polyphenol compound, CUR exerts a strong antioxidant effect. Therefore, the antioxidant activity of Man-CUR NPs in vitro was evaluated [35]. In brief, CLSM was applied to observe whether Man-CUR NPs could inhibit ROS production in RAW264.7 macrophages. The ROS level in RAW264.7 cells was detected by fluorescence probe DHE, emitting red fluorescence under oxidative condition. Distinct from the negative control, LPS-stimulated macrophages exhibited strong red fluorescence, indicating a large amount of ROS produced by inflammatory macrophages. Compared with the positive control, CUR NPs and Man-CUR NPs-treated cells showed weaker red fluorescence, particularly the Man-CUR NPs group (Fig. 4B), indicating the removal of ROS in large amounts from the cells. These results show that Man-CUR NPs can effectively reduce LPS-induced oxidative stress. To further investigate the effects of Man-CUR NPs on LPS-induced oxidative stress, an ELISA kit was used to detect ROS-related indicators. Both MDA and SOD levels were examined in the RAW264.7 cells treated with LPS (Fig. 4C). According to the results, MDA level increased and SOD level was reduced significantly after LPS stimulation (*p* < 0.01). After the incubation with NPs, the level of LPS-induced MDA in RAW264.7 cells decreased and that of SOD increased significantly, indicating the capability of Man-CUR NPs to scavenge oxygen free radicals and reduce oxidative stress.

Since CUR was found to drive macrophages to M2 polarization, the polarization of Man-CUR NPs were investigated in this study [15, 36, 37]. As shown in Figure S2, LPS-induced macrophages were photographed for verification. As shown in Fig. 4D, the expression level of M2 macrophage markers Mannose receptor (CD206) and Arginase (Arg-1) are lower in positive controls than in negative controls. Moreover, NPs treatment significantly enhances the expression level of CD206 and Arg-1, which indicates the suppressed transformation of macrophage into M2. These results demonstrate that Man-CUR NPs have the ability to drive macrophages into the M2 phenotype. In addition, Type M1 and Type M2 macrophages were induced by LPS and IL-4 respectively as positive control. The effects of different preparations on the expression of CD86 and CD206 were analyzed by flow cytometry. As shown in Fig. 4E, the expression of CD86 in macrophages treated with LPS increased significantly, while the expression of CD206 in macrophages treated with IL-4 increased significantly. Co-treatment of macrophages with LPS, Free CUR, CUR NPs and Man-CUR NPs decreased the expression of CD86 and increased the expression of CD206, and Man-CUR NPs showed a stronger effect of inhibiting the expression of CD86 and promoting the expression of CD206 than Free CUR. Therefore, the results showed that Man-CUR NPs could inhibit the polarization of macrophages to M1 type and promote the transformation of macrophages to M2 type.

To sum up, Man-CUR NPs show excellence in vitro anti-inflammatory, antioxidant activities and macrophage polarization. Their anti-inflammatory and anti-oxidative activities, and macrophage polarization are stronger than those without targeted modification. This is possibly attributed to the fact that the anti-inflammatory and anti-oxidative effects of CUR in macrophages are enhanced when the uptake of NPs is promoted by the targeting of Mannose receptor on the macrophage surface by Man.

### *In vivo* biodistribution evaluation

**Fig. 5:**
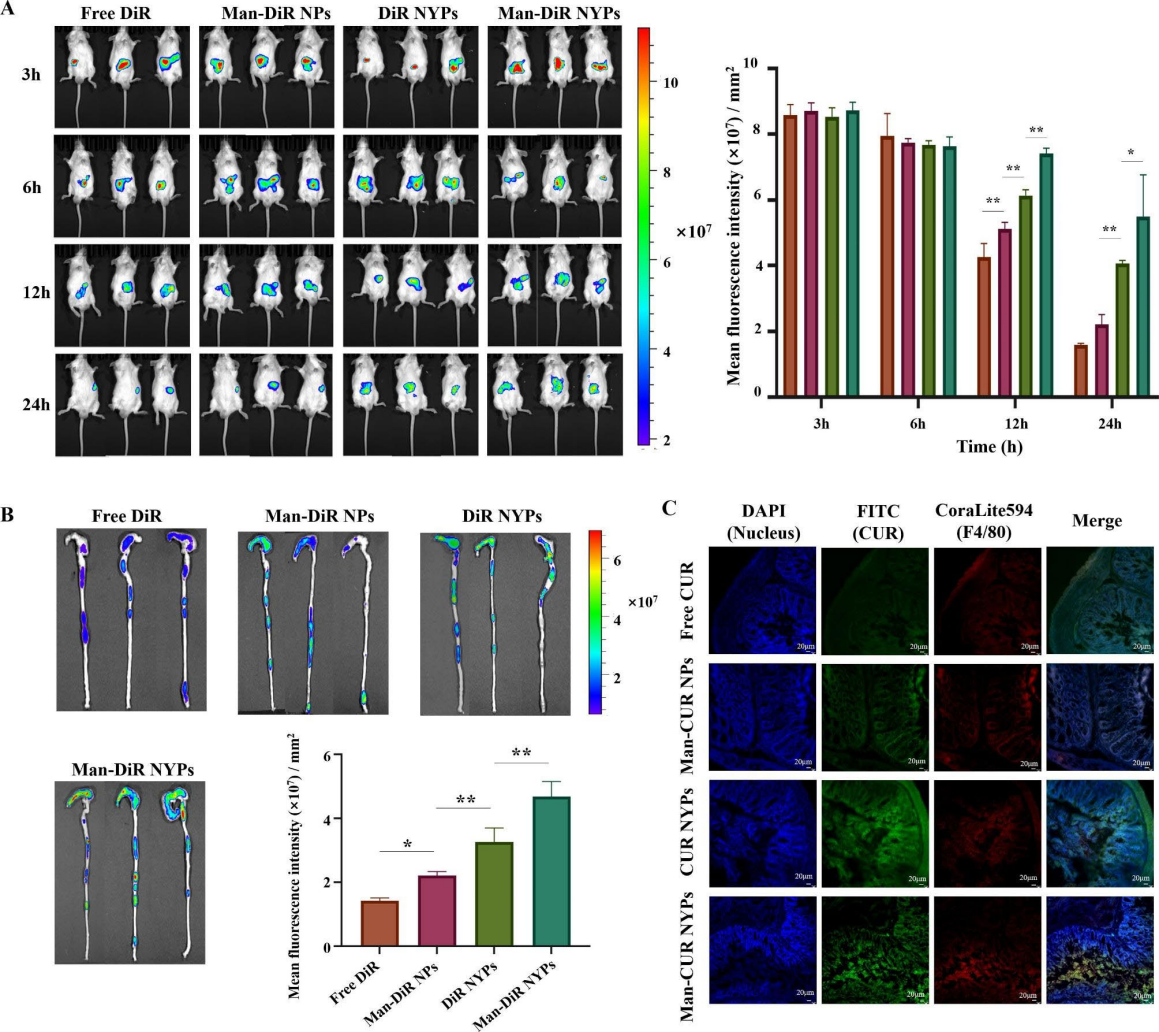
*In vivo* biodistribution of Man-CUR NYPs: (**A**) The mice fluorescence images and histograms at the time point of 3, 6, 12, and 24 h after oral administration of each formulation. (**B**) The colon fluorescence image and fluorescence signal histogram at the end point of each formulation. (**C**) Frozen sections. CUR/NPs (green); F4/80-macrophage (red); DAPI-nucleus (blue). Scale bar 20 μm. Data are mean ± SD (*n* = 3); *p < 0.05; **p < 0.01

The selective accumulation of drugs in colon tissue is one of the key factors that affect the treatment of UC by oral administration. In this study, IVIS and microscopy were adopted to assess the targeted distribution of Man-CUR NYPs in DSS-induced mice. After oral administration, fluorescence images were captured by an *in vivo* imaging system at specified time points. As shown in Fig. 5A, the variation in mean fluorescence intensity between different groups is insignificant after 3 or 6 h of oral administration. After 12 h, the mean fluorescence intensity of the Man-DiR NYPs reaches the highest level (*p* < 0.01), followed by DiR NYPs, Man-DiR NPs, and Free DiR. This situation lasts until the end of 24 h. Therefore, the changes of whole-body fluorescence in mice emphasized that YPs prolong drug retention in vivo. In addition, 24 h after oral administration, the colon was removed and photographed. The obtained fluorescence images and histograms are shown in Fig. 5B. According to the results, the mean fluorescence intensity around the colon in the Man-DiR NYPs group is higher than in the other groups (*p* < 0.01). That is to say, the NYPs constructed in this study extend the retention of the drug in colitis tissues and delay the removal of it in vivo, thus improving its bioavailability.

To further determine whether Man-CUR NPs are preferentially taken up by the macrophages in UC tissues, the colonic tissue frozen sections were stained with DAPI and coralite594 labeled F4/80 antibody to evaluate the capability of Man-CUR NYPs targeting macrophage *in vivo*. As shown in Fig. 5C, compared with Free CUR, more green fluorescence signals are detected in the colitis tissues of Man-CUR NPs-treated mice, with both green and red fluorescence showing evident colocalization. It is suggested that Man-CUR NPs could accumulate in colon tissue after oral administration, which is consistent with the result of cell uptake test. Furthermore, colon tissue exhibit stronger green fluorescence than NPs after NYPs treatment, suggesting that the encapsulation of YPs can improve the accumulation of NPs in colon tissue, which is possibly attributed to the protection of YPs by their intact access to colon sites. Furthermore, the green fluorescence signal of Man-CUR NYPs-treated colon is stronger than CUR NYPs-treatment is conducted, indicating the capacity of Man to improve the outcome of NPs targeting inflammatory colitis tissues. More importantly, green fluorescence and red fluorescence overlap well in Man-CUR NYPs-treated colonic tissues, suggesting that Man-CUR NYPs are highly selective for macrophages in the oral-administered tissues of inflammatory colitis. In addition, the selective distribution of Man-CUR NYPs in inflammatory colitis tissues is largely affected by the targeting ligand (Man) of NPs.

### *In vivo* therapeutic effect

#### Evaluation of ulcerative colitis commonly indicators

**Fig. 6:**
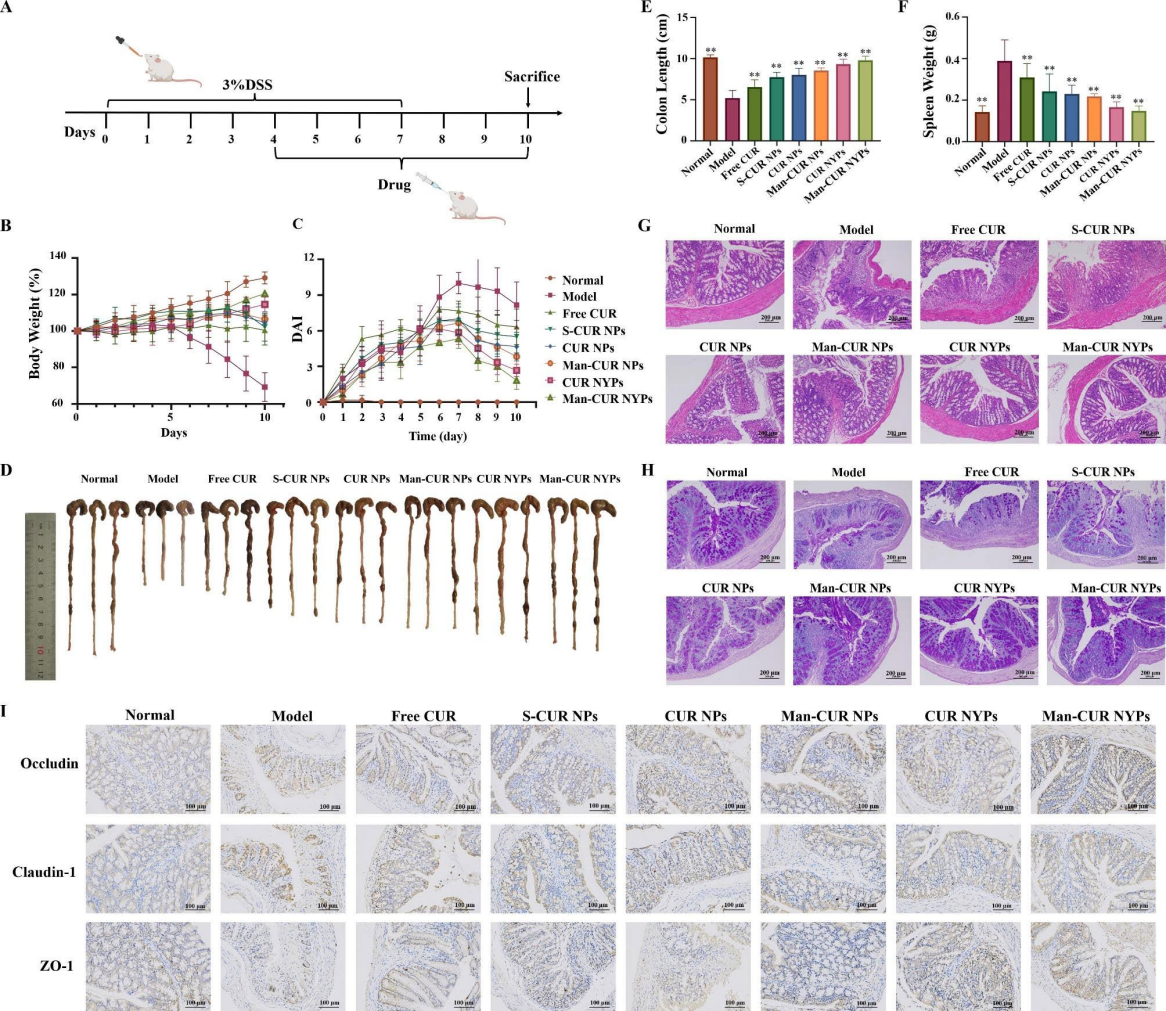
Commonly indicators of therapeutic effect of Man-CUR NYPs on UC mice: (**A**) Experimental design of drug treatment against UC. (**B**) Graph of body weight varying with time among different formulas. (**C**) Graph of DAI scores varying with time among different formulas. (**D**)Images of colon among different formulas. (**E**) Histogram of colon length. (**F**) Histogram of spleen index. (**G**) H&E-stained and (**H**) PAS-stained histopathological sections of the colonic tissues. (**I**) Representative IHC staining of Occludin, Claudin-1 and ZO-1 in the colon tissues. Data are mean ± SD (*n* = 6). *p < 0.05; **p < 0.01vs. Model

DSS-induced murine UC model was established to evaluate the therapeutic effect of Man-CUR NYPs on UC [38]. Figure 6 A illustrates the process of animal modeling and administration. On the fourth day after free drinking of 3% DSS, the mice developed typical symptoms of UC, such as diarrhea and blood in the stool. As shown in Fig. 6B, the body weight of mice in the model group decreases significantly from day 5, and it drops to nearly 69% of the initial level on day 10. The body weight of Man-CUR NYPs-treated mice shows an increasing trend compared with the other groups and it is most comparable to the normal group, suggesting the mitigating effect of Man-CUR NYPs on UC to some extent. As shown in Fig. 6C, the DAI score of Man-CUR NYPs and Man-CUR NPs groups shows a decreasing tend from day 7, which is lower compared to the Model group. On the last day, the DAI score of Man-CUR NYPs group is significantly lower than that of all the CUR NPs groups (none-PBAE CUR NPs (S-CUR NPs), CUR NPs and Man-CUR NPs) and Free CUR (*p* < 0.01), indicating the most significant effect of Man-CUR NYPs on UC. The similar results are obtained in the colon length (Fig. 6D, E), which shows that the colon is significantly shortened in the Model group. This is consistent with the clinical symptoms of UC. Moreover, Man-CUR NYPs demonstrated a greater effectiveness than all of the CUR NPs groups and Free CUR in maintaining colon length. This is similar to the normal group, highlighting the protective effect of YPs *in vivo*. Since the spleen can reflect how well the human immune system functions to some extent, the spleen index can be used to predict the severity of inflammation. As shown in Fig. 6F, the spleen index of the Man-CUR NYPs group is significantly lower compared to the Model group and other NPs treatment groups (*p* < 0.01). That is to say Man-CUR NYPs can reduce inflammation. H&E staining can reveal the inflammatory damage of colon tissue. Figure 6G shows the H&E staining results of colon tissue derived from the Model group mice, suggesting the severe damage caused to intestinal epithelial cells, the massive infiltration of inflammatory granulocytes and the damage suffered by the crypt structure. Both CUR NYPs and Man-CUR NYPs groups show mild lesions, which not only protects the crypt structure of colon but also reduces granulocyte infiltration and edema. It is further demontrated that Man-CUR NYPs can alleviate the morphological damage caused to colon by acute inflammation. Moreover, the PAS staining of colonic mucus goblet cells reveals the presence of goblet cells in the crypts of the normal group, while almost all goblet cells in the UC model group suffer severe damage (Fig. 6H). The results show that both CUR NPs and Man-CUR NYPs have protective effect on colon mucus goblet cells, and Man-CUR NYPs perform best in protecting on colon mucus goblet cells. This is consistent with the results of H&E staining. Finally, the main organ sections obtained from the treated mice show no histopathology evidence of damage (Figure S5A). And in the blood biochemical test of ALT, AST and BUN (Figure S5B), indicators of liver and kidney function, showed no difference from healthy mice. These demonstrate the high biocompatibility and low toxicity of the designed particles.

One of the most prominent pathological characteristics of DSS-induced colitis is the destruction of tight junction proteins in the intestinal epithelium [39]. Tight Junction (TJ) proteins, specially occludin, claudins and zonulaocculens (ZO), play a role in maintaining intestinal barrier function. The reduction of inflammatory response can lead to positive feedback of tight junction proteins. Therefore, immunohistochemistry was adopted to detect the expression level of claudin-1, occulin, and ZO-1 in colon tissues (Fig. 6I). It was observed that the expression levels of three proteins was suppressed in DSS-treated mice. Compared with the model group, the expression level of three proteins rose in the treatment group. Besides the Man-CUR NYPs group was comparable to the Normal group, indicating the repair effect of Man-CUR NYPs. These results show that Man-CUR NYPs can alleviate DSS-induced colitis to some extent by promoting intestinal mucosal barrier repair.

To sum up, the above results show that Man-CUR NYPs can effectively alleviate the common clinical symptoms of UC, reduce inflammatory reactions, and promote the repair of intestinal mucosal barrier, thus producing the best therapeutic effect on UC.

#### *In vivo* anti-inflammatory activity

**Fig. 7:**
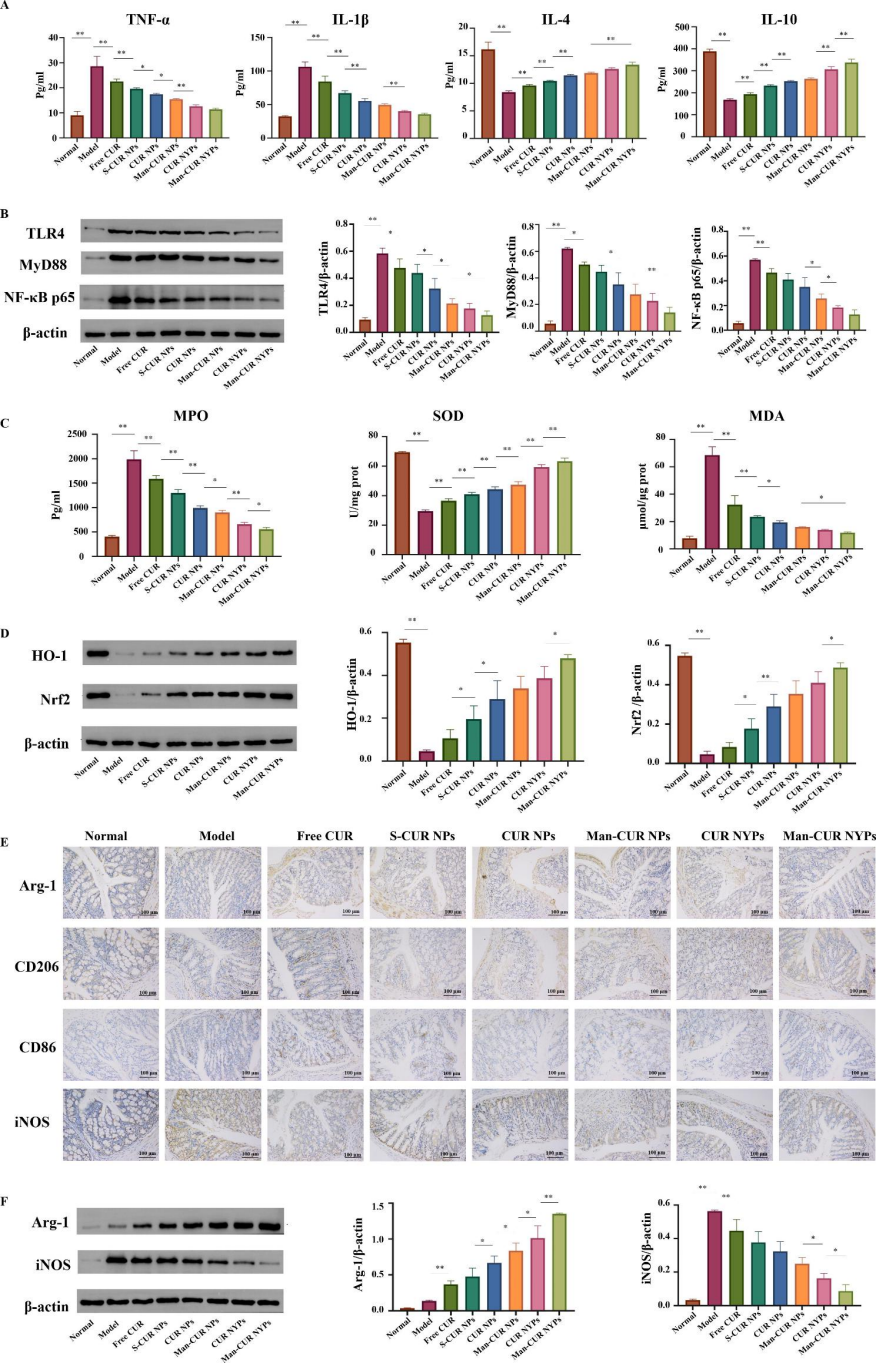
Anti-inflammatory and antioxidant effects of Man-CUR NYPs on UC mice* in vivo*: (**A**) The expression levels of TNF-α, IL-1β, IL-4 and IL-10 in the colons, measured by ELISA assays. (**B**) Western blotting results and histogram analysis of the influence of each group on the changes of TLR4, MyD88 and NF-κB p65 protein expression in colon. (**C**) The expression levels of MPO, SOD and MDA in the colons, measured by ELISA assays. (**D**) Western blotting results and histogram analysis of the influence of each group on the changes of HO-1 and Nrf-2 protein expression in colon. (**E**) Representative IHC staining of Arg-1, CD206, CD86 and iNOS in the colon tissues. (**F**) Western blotting results and histogram analysis of the influence of each group on the changes of Arg-1 and iNOS protein expression in colon. Data are mean ± SD (*n* = 6). *p < 0.05; **p < 0.01

In the previous *in vitro* study, it was found out that Man-CUR NPs can exert regulatory effects on inflammatory factors to some degree. In order to further explore the effect of Man-CUR NYPs on the inflammatory cytokines in inflammatory colon tissues, ELISA assay was performed to determine the content of inflammatory and anti-inflammatory cytokines in colon tissues. As shown in Fig. 7A, the level of pro-inflammatory cytokines TNF-α and IL-1β in the colon tissue of the Model group is significantly higher than in the Normal group (*p* < 0.01). After the treatment with Man-CUR NYPs, the level of TNF-α and IL-1β is significantly lower compared to the NPs treatment and Free CUR (*p* < 0.01). In addition, anti-inflammatory cytokines IL-4 and IL-10 show the opposite trend to TNF-α and IL-1β. CUR NYPs and Man-CUR NYPs show a significant difference in increasing IL-10 expression (*p* < 0.01), which indicated that Man could improve the targeting ability of nano-particles to some extent. This is consistent with the results of in vitro study. Besides, the expression of some inflammatory factors was observed. In order to further validate the mechanism of action, the associated proteins in the TLR4/NF-κB signaling pathway were investigated. TLR4 is an upstream regulator of NF-κB, the induction of which is critical to UC [40]. NF-κB is not only involved in the transcription of those genes related to inflammation and immune response, but also closely associated with the pathogenesis of UC [41]. TLR4 signaling plays an important role in activating the downstream inflammatory signaling pathways of NF-κB by recruiting myeloid differentiation factor 88(Myd88) and promoting the secretion of inflammatory cytokines [42]. In some studies, it has been shown that CUR can inhibit the over-expression of inflammatory mediators by blocking the TLR4/NF-κB signaling pathway [20]. According to the results of Western blotting the expression level of TLR4, Myd88 and NF-κB p65 proteins in the Model group is significantly higher than in the Normal group and the treated groups (Fig. 7B, p < 0.01). In addition, the expression level of the three proteins is reduced after drug treatment, with the Man-CUR NYPs group producing the best performance in inhibiting protein expression. The above results suggest that the inhibitiry effect of Man-CUR NYPs on the DSS-induced colitis in mice may be produced through the inhibition of those proteins related to TLR4/ NF-κB signaling pathway.

#### *In vivo* antioxidant activity

Closely related to oxidative stress, UC can trigger inflammation and affect the healing process of UC [43]. The MPO secreted by activated neutrophils is considered an important indicator as to the severity of UC. MDA and SOD are the significant indexes to detect used to assess lipid oxidative damage. Therefore, the contents of MPO, MDA and SOD in colitis tissues were detected by ELISA to evaluate the severity of colitis and the antioxidant effect of drugs [44]. As shown in Fig. 7C, the level of MPO and MDA in the colon tissue of the Model group is significantly increased (*p* < 0.01), while the activity of SOD is severely suppressed (*p* < 0.01). It means that the level of anti-oxidation in the colon of UC mice is significantly reduced and the oxidative damage is severe. Compared with Free CUR and NPs groups, the content of MPO and MDA in the colon tissue of Man-CUR NYPs group is significantly reduced (*p* < 0.01), while the activity of SOD is significantly enhanced (*p* < 0.01). These results suggest the role of Man-CUR NYPs in significantly improving the anti-oxidant activity of colon and mitigating the oxidative damage caused to colon. CUR NYPs and Man-CUR NYPs paly a significantly different role in reducing MPO content (*p* < 0.05) and increasing SOD activity (*p* < 0.01), indicating the possibility of Man enhancing the antioxidant capacity through targeted action. Based on our discovery of Man-CUR NYPs affecting oxidation-related indexes, it will be further studied through the oxidation-related pathway. The Nrf2/HO-1 signaling pathway is known as one of the key defense systems against oxidative stress [45]. According to some studies, CUR can inhibit oxidative stress by activating Nrf2/HO-1 pathway [46]. Therefore, an analysis was conducted as to the effect of Man-CUR NYPs on the Nrf2/HO-1 signaling pathway. As shown in Fig. 7D, the expression of Nrf2 and HO-1 protein is significantly decreased in the Model group (*p* < 0.01). Compared with the CUR NPs, Man-CUR NYPs activate the Nrf2/HO-1 signaling pathway more significantly (*p* < 0.01). That is to say, the use of CUR to deliver YPs for UC treatment can produce antioxidant effect on the colon.

#### *In vivo* macrophage reprogramming

Previous *in vitro* studies have demonstrated that Man-CUR NPs may induce the transformation of macrophages into M2 phenotype. Therefore, in order to further explore the regulatory effect of Man-CUR NYPs on macrophage polarization, the expression of CD86, iNOS, CD206 and Arginase-1(Arg-1) in the colon of mice was determined by immunohistochemistry (IHC) [47]. As shown in Fig. 7E, compared with the Model group, the expression of CD86 and iNOS in the administration group is reduced, and Man-CUR NYPs showed a better effect. The expression of CD206 and Arg-1 in colon tissue enhanced after treatment, especially in the Man-CUR NYPs group. That is to say, CD86 and iNOS (M1 marker) decreased while CD206 and Arg-1 (M2 marker) increase in colon tissue, indicating the potential of Man-CUR NYPs to transform macrophage from M1 to M2 phenotype. It was observed that Man-CUR NYPs exerted a regulatory effect on M1 and M2 related markers. In order to quantify polarization-related proteins, the protein level of iNOS and Arg-1 in colon tissue was further detected by Western blotting (Fig. 7F). The results show that Man-CUR NYPs could up-regulate the expression of Arg-1 and down-regulate the expression of iNOS. According to the above results, Man-CUR NYPs can induce the transformation of macrophages from M1 to M2 in inflammatory tissues and improve the inflammatory level.

The above results indicate that Man-CUR NYPs have a significant improvement effect on the symptoms of UC mice. Although some indicator results showed no significant difference between CUR NYPs and Man-CUR NYPs. However, there are significant differences in macrophage polarization and antioxidant related indicators. In conclusion, Man-CUR NYPs can effectively treat UC mice through the synergistic effect of multiple effects.

## Conclusion

In summary, we constructed a promising strategy for “supramolecular curcumin nanoparticles with pH/ROS sensitive and multistage therapeutic effects” in “advanced yeast particles”. YPs can target delivery Man-CUR NPs to the inflammatory lesions and protect Man-CUR NPs against environmental assaults to improve the stability of Man-CUR NPs in oral delivery. As revealed by SEM and CLSM, YPs are transformed from a porous hollow structure to a smooth and full spherical structure, which indicates that Man-CUR NPs can enter and fill the core through the pores on the surface of YPs. Notably, through a study on the stability of Man-CUR NYPs, SEM results are obtained to indicate that YPs can pass through the gastric environment and break down under the action of colonic microbes or enzymes which facilitates the smooth delivery of Man-CUR NPs to the colon target site. In addition, to verify the functionality of supra-NPs, *Iin vitro* uptake experiments results showed that D-Man could improve the efficiency of NPs internalization into macrophages. On the other hand, dual responsive nanomaterials are constructed with HPβCD as the structural unit, which enables the encapsulation of CUR and pH/ROS dual-sensitive response. Interestingly, the results show that the EE% of CUR increases by about 90.24%±1.49%. The morphology of NPs shows changes in the macrophage environment simulated with different pH values and ROS and pH/ROS double-sensitive NPs exhibit more fast release behavior than single-sensitive NPs. Furthermore, the pharmacodynamics of CUR-loaded supra-NPs and NYPs are studied both *in vitro* and *in vivo*. CUR-NPs also enhanced LPS induced inflammatory model in macrophages through downregulation of pro-inflammatory factors, upregulation of anti-inflammatory factors, M2 macrophage polarization, and scavenging the excess ROS. Moreover, the results of *in vivo* experiments showed that Man-CUR NYPs can exert anti-inflammatory effects on both commonly indicators and inflammatory-related factors, verified by TLR4/NF-κB signaling pathway. In addition, Man-CUR NPs are found capable to induce the elevation of macrophage M2 phenotype *in vitro* studies. What’s more, Man-CUR NYPs can polarize macrophages from M1 to M2 in vivo macrophage polarization experiments. Finally, both Man-CUR NPs and Man-CUR NYPs show significant antioxidant effects. Further studies show that Man-CUR NYPs could regulate the expression of antioxidant enzymes HO-1 and Nrf2 by activating the Nrf2/HO-1 signaling pathway. These results collectively suggest that this “supramolecular nanoparticles” in “advanced yeast particles” novel platform with versatile pharmacological functions can be used as an effective tool for UC therapy via oral administration.

### Electronic supplementary material

Below is the link to the electronic supplementary material.

Supplementary Material 1

## Data Availability

The data and material are available.
