# Supplementary material for: “Dual sensitive supramolecular curcumin nanoparticles” in “advanced yeast particles” mediate macrophage reprogramming, ROS scavenging and inflammation resolution for ulcerative colitis treatment
Source: J Nanobiotechnology. 2023 Sep 7;21:321. doi: 10.1186/s12951-023-01976-2 (PMC10483867; doi:10.1186/s12951-023-01976-2)
Supplement: Supplementary file 1 — Supplementary Material 1 [file 12951_2023_1976_MOESM1_ESM.docx]

**Supplementary** **Materials**

**“Dual Sensitive Supramolecular Curcumin Nanoparticles” in “Yeast Particles” Mediate Macrophage Reprogramming, ROS Scavenging and Inflammation Resolution for Ulcerative Colitis Treatment**

*Xiaoqin Han* ^a,1^, *Ruifeng Luo* ^a,c,1^, *Shanshan Qi* ^a^, *Yanli Wang* ^a^, *Linxin Dai* ^a^, *Wenbiao Nie* ^a^, *Meisi Lin* ^a^, *Haoqi He* ^a^, *Naijing Ye* ^b^, *Chaomei Fu* ^a^, *Yu You* ^a*^, *Shu Fu* ^a*^, *Fei Gao* ^a*^

^a^State Key Laboratory of Southwestern Chinese Medicine Resources, Pharmacy School, Chengdu University of Traditional Chinese Medicine, Chengdu 611130, China

^b^ TCM Regulating Metabolic Diseases Key Laboratory of Sichuan Province, Hospital of Chengdu University of Traditional Chinese Medicine, Chengdu, 610072, China

^c^ State Key Laboratory of Quality Research in Chinese Medicine, Institute of Chinese Medical Sciences, University of Macau, Taipa, Macau 999078, China.

Corresponding author: Fei Gao; Shu Fu; Yu You; Meisi Lin

*Email: feigao207@yeah.net; [61769335@qq.com](mailto:61769335@qq.com); [20122071@cdutcm.edu.cn](mailto:20122071@cdutcm.edu.cn); 1067584380@qq.com

^1^ These authors are equal contribution to this work.


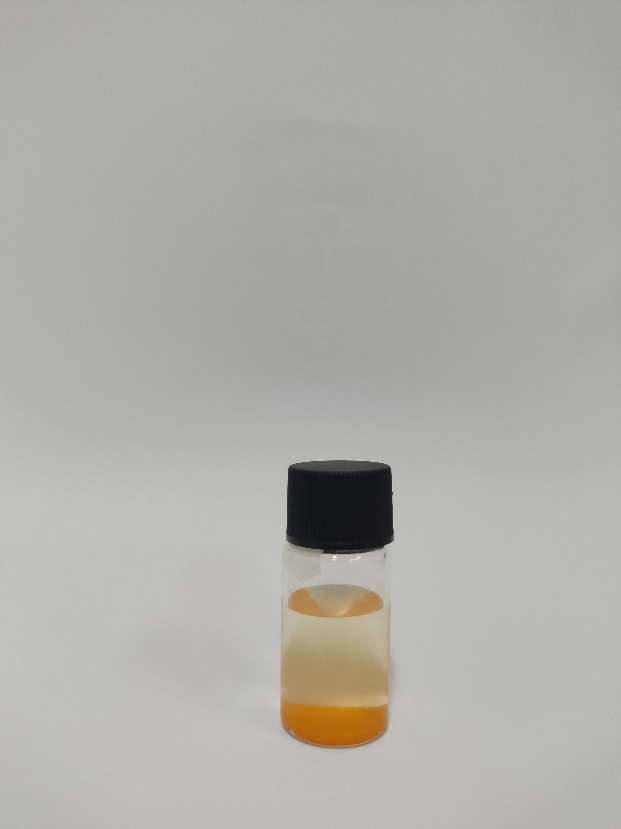


**Figure S1.** The appearance of CUR coated with HPβCD alone.


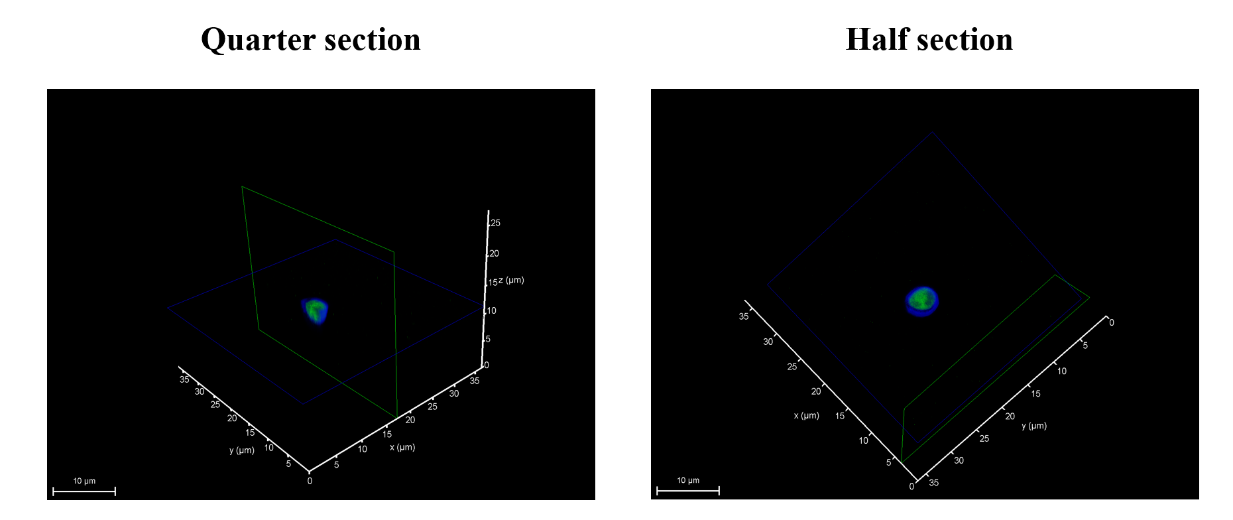


**Figure S2.** Representative CLSM 3D images of Man-CUR NPs (green) and the yeast cell wall was labeled with calcofluor white (blue; scale bar = 10 μm).

**
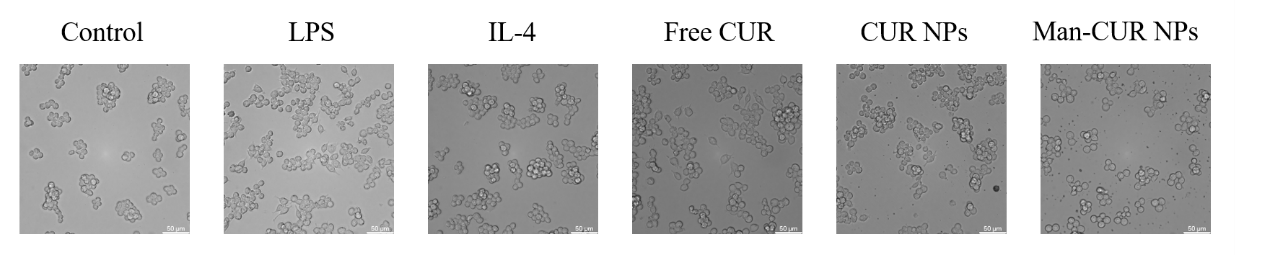
**

**Figure S3.** RAW264.7 cells were polarized into M1 or M2 and incubated with various NP preparations during the polarization process.


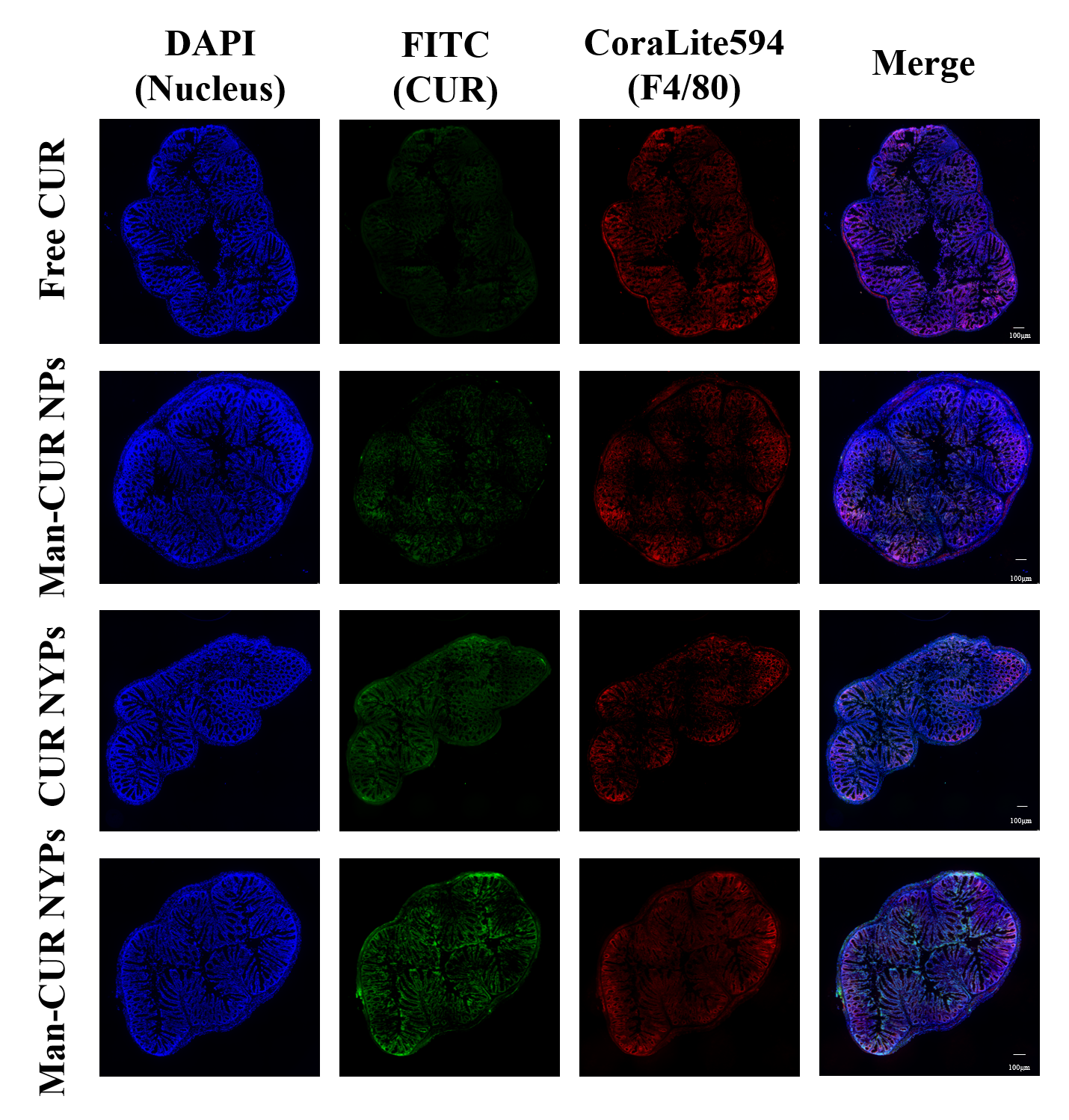


**Figure S4.** Full scan frozen section image of colon. CUR/NPs (green); F4/80-macrophage (red); DAPI-nucleus (blue). Scale bar 100 μm.


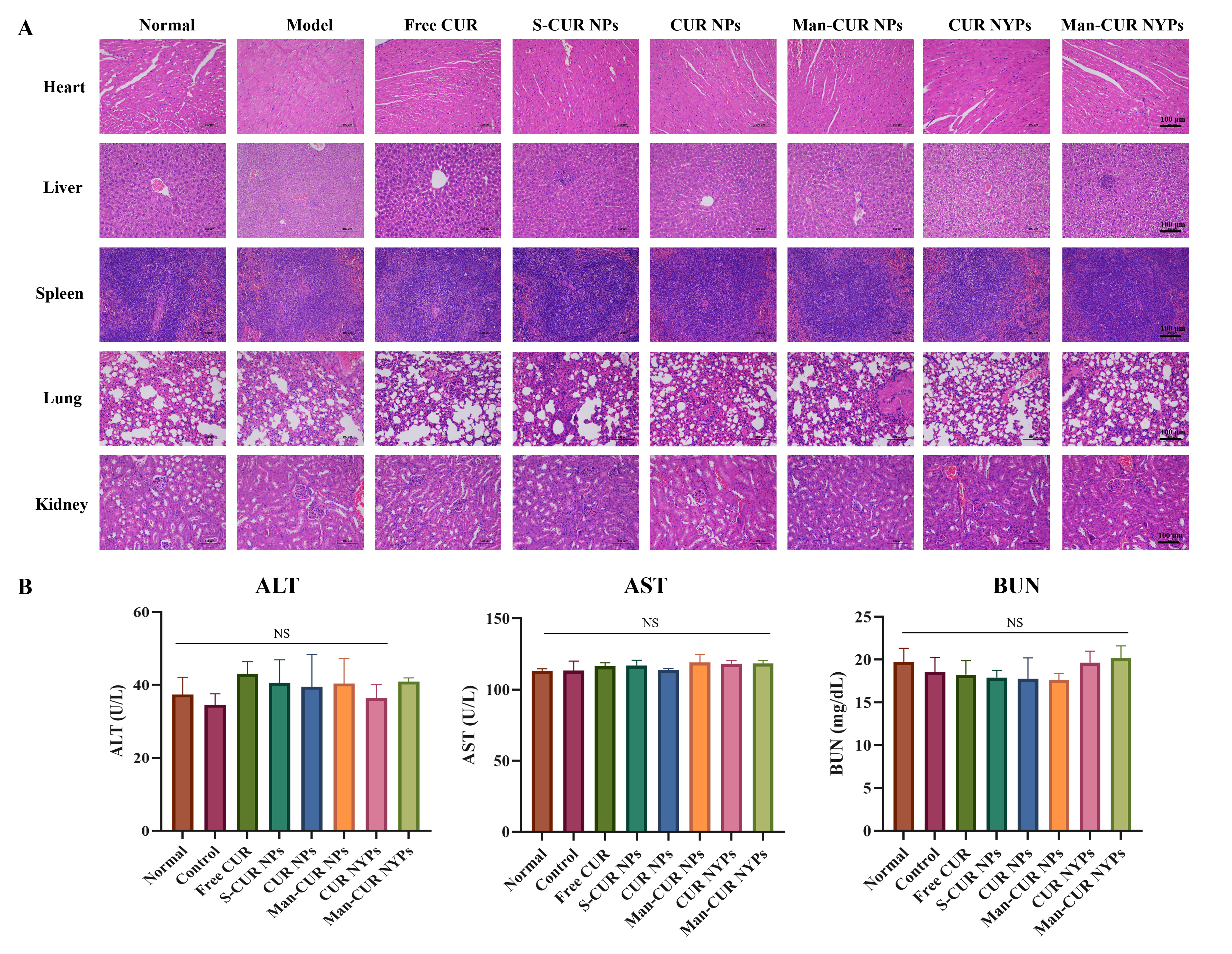


**Figure S5.** Safety evaluation: (A) H&E staining of heart, liver, spleen, lung, and kidney. The scale bar represents 100 μm. (B) Blood biochemical test of ALT, AST and BUN. NS, no significance.

**Table S1.** Characteristic of Man-CUR NPs

|  | Average size (nm) | Polydispersity Index  (PDI) | ζ- potential  (mV) | EE  (%) | LE  (%) |
| --- | --- | --- | --- | --- | --- |
| Man-CUR NPs | 140.58  145.26  144.49 | 0.189  0.163  0.115 | 16.6  16.3  16.8 | 92.32  89.49  88.90 | 8.46  8.51  8.65 |
